# Evaluation of the reactogenicity, adjuvanticity and antigenicity of LT(R192G) and LT(R192G/L211A) by intradermal immunization in mice

**DOI:** 10.1371/journal.pone.0224073

**Published:** 2019-11-04

**Authors:** Milton Maciel, Mark Smith, Steven T. Poole, Renee M. Laird, Julianne E. Rollenhagen, Robert W. Kaminski, Heather Wenzel, A. Louis Bourgeois, Stephen J. Savarino

**Affiliations:** 1 Henry M. Jackson Foundation for the Advancement of Military Medicine, Bethesda, MD, United States of America; 2 Department of Microbiology and Immunology, Uniformed Services University of the Health Sciences, Bethesda, MD, United States of America; 3 Enteric Diseases Department, Naval Medical Research Center, Silver Spring, MD, United States of America; 4 Subunit Enteric Vaccines and Immunology, Walter Reed Army Institute of Research, Silver Spring, MD, United States of America; 5 PATH, Washington, D.C., United States of America; 6 Department of Pediatrics, Uniformed Services University of the Health Sciences, Bethesda, MD, United States of America; New York State Department of Health, UNITED STATES

## Abstract

The development of an effective subunit vaccine is frequently complicated by the difficulty of eliciting protective immune responses, often requiring the co-administration of an adjuvant. Heat-labile toxin (LT), an enterotoxin expressed by enterotoxigenic *E*. *coli* (ETEC) with an AB_5_ structure similar to cholera toxin, is a strong adjuvant. While the mucosa represents the natural route of exposure to LT and related toxins, the clinical utility of LT and similar adjuvants given by mucosal routes has been limited by toxicity, as well as the association between intranasal delivery of LT and Bell’s palsy. Single and double amino acid mutants of LT, LT(R192G)/mLT and LT(R192G/L211A)/dmLT respectively, have been proposed as alternatives to reduce the toxicity associated with the holotoxin. In the present study, we compared mLT and dmLT given via a non-mucosal route (i.e. intradermally) to investigate their adjuvanticity when co-administrated with an enterotoxigenic *E*. *coli* vaccine candidate, CfaEB. Antigenicity (i.e. ability to elicit response against LT) and reactogenicity at the injection site were also evaluated. BALB/c mice were immunized by the intradermal route with CfaEB plus increasing doses of either mLT or dmLT (0.01 to 2.5 μg). Both adjuvants induced dose-dependent skin reactogenicity, with dmLT being less reactogenic than mLT. Both adjuvants significantly boosted the anti-CfaE IgG and functional hemagglutination inhibiting (HAI) antibody responses, compared to the antigen alone. In addition to inducing anti-LT responses, even at the lowest dose tested (0.01 μg), the adjuvants also prompted *in vitro* cytokine responses (IFN-γ, IL-4, IL-5, IL-10 and IL-17) that followed different patterns, depending on the protein used for stimulation (CfaE or LTB) and/or the dose used for immunization. The two LT mutants evaluated here, mLT and dmLT, are potent adjuvants for intradermal immunization and should be further investigated for the intradermal delivery of subunit ETEC vaccines.

## Introduction

Live attenuated and inactivated vaccine platforms, whether bacterial or viral, are typically highly immunogenic. In contrast, recombinant protein-based vaccines often have limited antigenicity due the lack of immune danger signals, requiring an adjuvant to promote adequate immune response. The heat-labile toxin (LT) from the enterotoxigenic *E*. *coli* (ETEC) bacterium has been investigated as an adjuvant for delivery of antigens via mucosal routes, but due to its inherent toxicity to promote diarrhea, as well as by cases of Bell’s palsy observed after vaccination with an influenza vaccine delivered intranasally with LT, its use was discontinued [1]. Efforts to delink toxicity from adjuvanticity through modifications of LT, such as incorporating one or more amino acid substitutions, resulted in proteins with reduced or absent enterotoxicity and different adjuvant properties [2–4]. The most promising of these are LT(R192G), a single mutant of LT (mLT) and LT(R192G/L211A), a double mutant of LT (dmLT). Both are potent adjuvants when given orally [5–9] and have diminished toxigenic profiles [4, 10].

We have been interested in using mLT and dmLT as adjuvants for the delivery of a subunit anti-ETEC vaccine since both adjuvants can also elicit immune responses against LT, which has been suggested as protective against diarrhea caused by LT^+^ ETEC [11]. Our ETEC vaccine strategy incorporates both the potential for toxin neutralization and the inhibition of colonization through the disruption of bacterial adherence to the intestinal epithelium. For the latter, we generated recombinant subunit antigens from ETEC colonization factors (CFs) such as CfaE, the minor fimbrial adhesin of the CFA/I CF [12] and the dimeric fusion CssBA, which contains the CssA and CssB subunits of CS6 [13]. These recombinant antigens remain immunogenic and able to promote functional antibodies, capable of inhibiting bacterial binding to red blood cells in the case of class 5 CF-derived antigens (i.e. CfaE) [5], or to intestinal cell lines, in the case of CS6 antigens [13]. CfaE, our prototype class 5a ETEC antigen, has been tested in mice and *Aotus nancyamaae* non-human primates, where it was immunogenic when given orally or intranasally with mLT as the adjuvant [5] and protected the monkeys from diarrhea after oral challenge with CFA/I^+^ ETEC strain H10407 [14]. In addition, volunteers either orally treated with bovine hyper immunoglobulin raised against CfaE or actively vaccinated intradermally with CfaE + mLT were protected from diarrhea after oral challenge with H10407 [15, 16].

Dermal vaccination has a proven track record [17–19] and important advantages: it is less painful than intramuscular injections, is dose sparing, and uses lower volumes. The structure of the skin is such that antigens applied into the skin gain direct access to the network of antigen-presenting cells, such as Langerhans and dendritic cells, which can readily initiate an immune response [20, 21]. Moreover, as experimentally demonstrated for transcutaneous immunization (TCI), when the antigen is applied onto the skin, mucosal immune responses can also be achieved [22, 23]. While it is well established that the intradermal toxicity of the LT holotoxin is directly dependent on the capacity of the B subunit to bind host cell membrane receptor ganglioside GM1 [24, 25], the effect of mutations in the LTA subunit has not been properly investigated. Here, we evaluated the use of mLT and dmLT as adjuvants for the intradermal delivery of a recently developed ETEC antigen fusion, CfaEB, which is a combination of the minor adhesin, CfaE, and the major pilin, CfaB, of the CFA/I CF. We demonstrated that both adjuvants were suitable for intradermal immunization, had a benign and transient local reactogenicity profile, which subsided over a few weeks, enhanced the immune response against the co-administered antigen, CfaEB, and promoted anti-toxin response.

## Materials and methods

### Vaccine antigens

The *Escherichia coli* single mutant heat-labile toxin [LT(R192G), hereafter mLT] was cloned, expressed and purified from *E*. *coli* strain JM83(pLC326) at Walter Reed Army Institute of Research (WRAIR) Pilot Bioproduction Facility (BPR 466–00 lot 0816, Forest Glen Annex, Silver Spring MD). The *Escherichia coli* double-mutant heat-labile toxin [LT(R192G/L211A), hereafter dmLT] was produced at WRAIR Pilot Bioproduction Facility (BPR-1037-00, Lot #1735), following previously described affinity-chromatography methods [26], and obtained through PATH (Washington D.C.). Since we and others [26] have previously observed that low concentrations of mLT and dmLT tend to get absorbed to polypropylene and borosilicate vials, doses were prepared in Daikyo Crystal Zenith® vials (West Pharmaceutical Services, Exton, PA). Immediately after administration of the first dose, the presence of mLT or dmLT in the formulations was assessed by Western blot with anti-LT Ab. The amount of mLT or dmLT, judged by the intensity of the subunit B band, approximately corresponded to the expected concentration of the respective protein in each group’s dose (S1A Fig). At the highest dose used (2.5 μg), mLT and dmLT endotoxin content was ≤ 0.15 EU/dose.

The donor strand-complemented (dsc) CfaEB fusion protein (hereafter CfaEB), expressing the minor (CfaE) and major (CfaB) subunits of CFA/I colonization factor (CF), was expressed and purified from a BL21(DE3) expression host using previously described methods [27]. Briefly, CfaEB was purified from cell lysates using a HisTrap FF column, eluting the protein with a linear imidazole gradient (5–300 mM Imidazole over 20 column volumes) in a 20 mM phosphate buffer pH 7.4, followed by a HiTrap SP HP column, eluting the protein with a linear salt gradient (0–1 M sodium chloride) in a 20 MES buffer, pH 5.5. The concentration of CfaEB in the vaccine preparations was assessed by densitometry measurements on formulation samples separated by SDS-PAGE. The intensity of the CfaEB band in the formulation samples was then determined using a standard curve constructed from the band intensities of known CfaEB standards separated on the same gel. All CfaEB doses were within ± 20% of the target dose of 10 μg (S1B Fig). The dose of 10 μg of CfaEB contained 0.18 EU/dose of endotoxin.

### Immunization and sample collection

**Fig 1:**
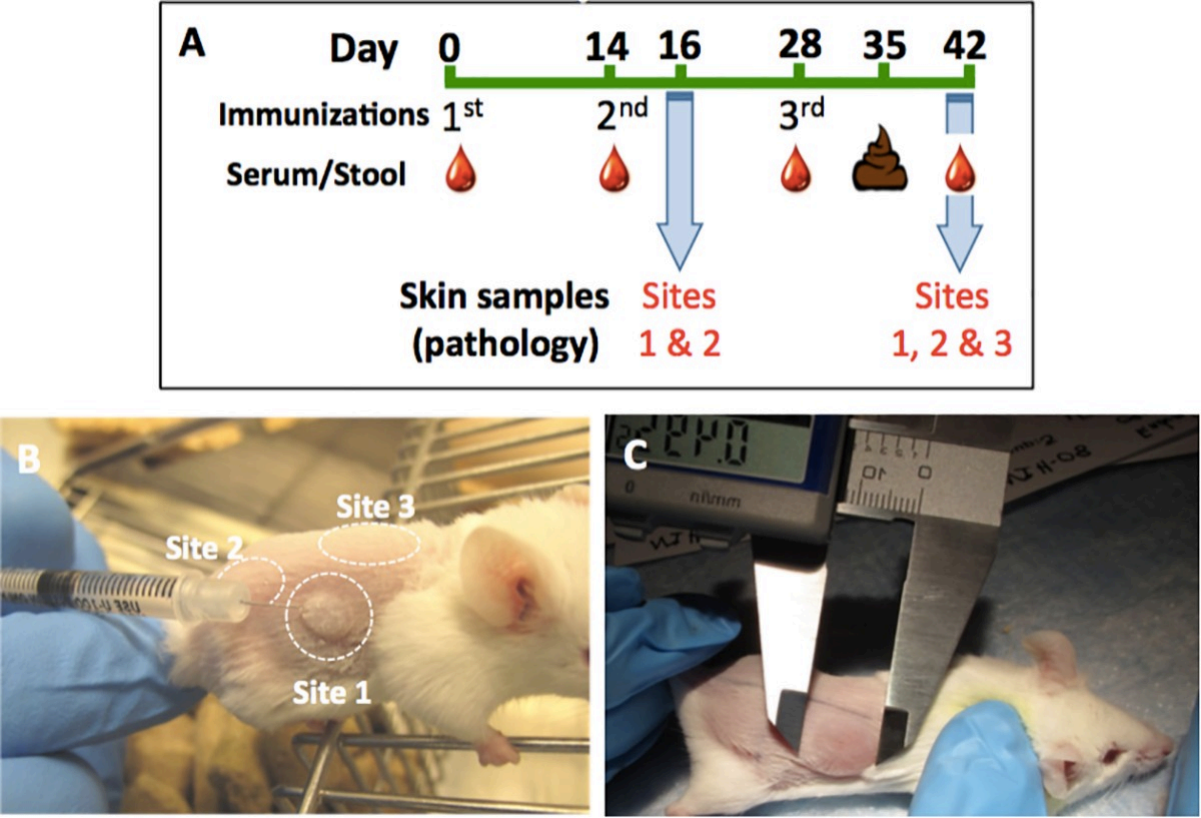
Study design and measurement of skin induration. **(A)** BALB/c mice (n = 5-7/group) were immunized intradermally on days 0, 14, and 28. Blood was obtained on days 0, 14, 28 and 42, and fecal samples were obtained on day 35. In a subset of mice, pathology biopsies were taken from immunization site 1 (S1) and immunization site 2 (S2) on d16, and from S1, S2 and immunization site 3 (S3) on d42. **(B)** Mice were shaved on the dorsum and immunized on days 0, 14, and 28 at S1, S2 and S3, respectively. **(C)** Induration was measured (in mm) using a digital caliper by length (caudal to cranial) and width (medial to lateral) and then the two measurements were averaged.

Animal studies were approved by the Institutional Animal Care and Use Committee at the Naval Medical Research Center. Female BALB/c mice, ages 6–8 weeks (The Jackson Laboratory, Bar Harbor, Maine) were housed in laminar flow cages for 7 days before use. Food and water were provided *ad libitum*. Intradermal (ID) immunizations were performed on day 0 (d0), d14 and d28 (Fig 1A). One day before the immunization, the mice were restrained and the dorsa shaved. On the day of immunization, animals were manually restrained and 20 μL of the vaccine was delivered using a 31G needle fitted to a 1 ml syringe inserted almost parallel to the skin. A bleb at the injection site indicated the proper intradermal delivery of the vaccine (Fig 1B). The three doses were administered at a different site on the animal’s dorsum (Fig 1B) in order to allow independent measurement of skin reactogenicity. Twelve groups of seven mice were immunized as described in Table 1. Blood was sampled at baseline (d0), and two weeks after each immunization (d14, d28 and d42). Samples collected up to and including d28 were collected by tail bleeding non-anesthetized animals (Fig 1A). On d42, animals were anesthetized with Ketamine-Xylazine (Phoenix Scientific, Inc., St. Josephs MO), exsanguinated by cardiac puncture with a 25-gauge needle and 1cc syringe, and euthanized by cervical dislocation. Blood was centrifuged at 400g at 4°C for 15 min, and serum stored at -20°C. Fecal pellets, 3-5/animal, were collected on d35 and processed in Protease Inhibitor Cocktail (P2714; Sigma-Aldrich, Saint Louis, MO), and stored at -20°C.

**Table 1:** Groups’ formulations.

Group	Doses (μg)		
	mLT	dmLT	CfaEB
1	2.5	-	10
2	0.5	-	10
3	0.1	-	10
4	0.05	-	10
5	0.01	-	10
6	-	2.5	10
7	-	0.5	10
8	-	0.1	10
9	-	0.05	10
10	-	0.01	10
11	-	-	10
12	Saline		

### Skin reactogenicity

As a primary parameter of local reactogenicity to the ID injections, induration was measured at 24, 48, and 72 h after each immunization, and every 7 days thereafter until resolution or the end of the study. Animals were restrained, and palpable local induration was measured with the aid of an electronic caliper (VWR, Philadelphia, PA) and recorded in millimeters (mm) (Fig 1C). The diameter of the induration was measured by length (caudal to cranial) and width (medial to lateral), and averaged for analysis and graphic presentation. In parallel, adapted Draize scores [28] were also recorded according to the scale in Table 2.

**Table 2:** Adapted Draize scale.

Score	Grade	Edema	Erythema
0	None	No swelling	Normal color
1	Minimal	Slight swelling (barely perceptible)	Light pink (barely perceptible)
2	Mild	Defined swelling (distinct)	Bright pink/pale red
3	Moderate	Defined swelling (raised ≤ 1 mm)	Bright red
4	severe	Pronounced swelling (> 1 mm)	Dark red

### Skin pathology

Skin samples from the injection sites were collected on two occasions, on d16 and d42 (see Fig 1A). Two mice from each group were euthanized on d16 after the first immunization and skin samples of approximately 1.5 cm^2^ from injection site 1 (S1; 16 days after the first immunization) and site 2 (S2; two days after second immunization) were collected for histologic analysis. At the end of the study, skin samples were again collected from two mice per group. Specifically, skin was collected from S1 (42 days after immunization), S2 (28 days after immunization) and site 3 (S3; 14 days after the last immunization). Skin samples were placed in 10% neutral buffered formalin and fixed for a minimum of 24 h at room temperature (RT). The fixed tissue was processed by ethanol dehydration and embedded in paraffin, then cut into 5 μm thick sections and stained with hematoxylin and eosin (H&E). The H&E sections of the skin were evaluated in a blinded manner by a board-certified veterinary pathologist. Inflammation in the skin was characterized and assigned a severity score which ranged from 0 to 5 as follows: 0, no lesions; 1, minimal; 2, mild; 3, moderate; 4, marked; and 5, severe.

### Analysis of antibodies in mouse serum samples by ELISA

Sera and stool extracts were assessed for anti-CFA/I, anti-CfaE, and anti–LTB IgG (total and IgG subclasses) and IgA antibody (Ab) titers by ELISA. CfaE [5, 29] and recombinant LTB (B subunit of LT) were manufactured in our laboratories. Assays were performed with individual serum samples, except where otherwise indicated. Antigen (Ag)-specific IgG ELISA was performed on Nunc^™^ MicroWell^™^ (Thermo Scientific, Rochester, NY), while Ag-specific IgA assays were performed on Nunc^™^ MicroWell^™^ Maxisorp^™^ 96-well plates (Thermo Scientific, Rochester, NY). For anti-CfaE-specific assays, plates were coated with CfaE at 1.0 μg/mL (IgG) or 2.0 μg/mL (IgA) in PBS, while plates for LTB-specific assays were first coated with GM1 (Sigma-Aldrich) at 0.5 μg/mL. Both were coated with a volume of 100 μL/well, for 1 h at 37°C (IgG) or overnight at 37°C (IgA), followed by overnight (ON) at 4°C. After three washes with PBS, all plates were blocked with 150 μl/well (IgG) or 200 μl/well (IgA) of 5% non-fat milk (Sigma-Aldrich) in 0.05% Tween-20 (Sigma-Aldrich)-PBS (PBS-T) for 1 h at 37°C in a humidified chamber. Recombinant LTB was added to LTB-specific plates at 0.5 μg/mL and incubated at 37°C for 1 h. After three washes with PBS-T, serum samples were added at a starting dilution of 1:50 in 1% non-fat milk-PBS-T followed by a 3-fold serial dilution, and incubated for 1.5 h at RT. Plates were washed 5 times with PBS-T followed by addition of 0.8 μg/mL peroxidase-conjugated goat anti-mouse (H+L) IgG (KPL, Gaithersburg, MD), 1:100 dilution of peroxidase-conjugated rat anti-mouse IgG1 and IgG2a) (Bethyl Laboratories, Montgomery, Texas) or 0.5 μg/mL peroxidase-conjugated goat anti-mouse IgA (KPL) in 1% non-fat milk-PBS-T for 1.5 h at 37°C in a humidified chamber (IgG) or RT (IgG subclasses and IgA). For IgA assays, plates were washed and 1-Step Ultra TMB (3,3',5,5'-tetramethylbenzidine; Fisher Scientific, Waltham, Massachusetts) was added at 100 μL/well and incubated for 30 min at RT. TMB Stop solution (KPL) was added at 50 μL/well and incubated at RT for 2 min. For IgG assays, plates were washed and Orthophenylenediamine + hydrogen peroxide (Sigma-Aldrich) in Sodium Citrate Buffer (Sigma-Aldrich) was added at 100 μL/well and incubated for 20 min at RT. After incubations, optical density (OD) was measured at 450 nm using a Multiskan EX^®^ ELISA reader with Ascent^®^ software (Thermo Scientific), which calculated the final Ab titers. The cut-off for each plate was calculated by the average of the background wells OD plus a fixed value of 0.4 for total IgG and IgA, or 3 times the background level for IgG1 and IgG2a. Fecal extracts were assayed by ELISA for anti-CfaE and anti-LTB IgA as described above. A linear regression was fitted to the experimental data, and the endpoint titer was determined as the reciprocal of the interpolated sample dilution that intersected with the cutoff, and log_10_-transformed. The average log_10_ titer for duplicates was calculated as the final result. Serum samples with OD below the cutoff, even at the top serum dilution, were assigned a value of one-half of the lowest dilution tested (i.e. 1:25) for computational purposes.

### Hemagglutination inhibition assay

The hemagglutination inhibition (HAI) assay, an adaptation of the mannose-resistant hemagglutination assay (MRHA) [30], was performed with mouse sera samples to assess the level of functional antibodies (Abs). Minimum Hemagglutination Titer (MHT), defined as the reciprocal of the lowest dilution of bacteria that agglutinated bovine red blood cells (BRBC), was determined daily. Briefly, CFA/I^+^ ETEC (strain H10407) bacteria were grown ON on CFA agar plates with bile salts, harvested, and resuspended in 0.5% D-mannose (Sigma-Aldrich) in PBS (PBS-M) to a final solution with OD_650_ 0.2 ± 0.02. Twenty-five μL of this suspension was added to wells of microtiter plates (Falcon Microtest^™^ U-bottom tissue culture treated, BD, Franklin Lakes, NJ), followed by 2-fold serial dilution with PBS-M. Twenty-five μL of 1.5% BRBC (Lampire Laboratories, Pipersville, PA; stored up to 10 days at 4°C in Alsever’s solution prior to use) in PBS-M plus 25 μL of PBS-M was added to each dilution. Plates were shaken at 0.5 RPM for 30 min at 4°C, and agglutination was assessed. The highest dilution of bacteria to give visible agglutination was taken as the MHT for the day. The HAI assay was performed with a bacteria suspension two dilutions more concentrated than the one defined as MHT. For the HAI assay, 25 μL of the serum samples were diluted 1:8 with PBS-M and added to the wells, followed by 2-fold serial dilution (up to 1:16,384) in PBS-M. The suspension of bacteria was added in equal volume, and the plate was shaken at 500 RPM for 30 min at RT. After the incubation, 25 μL of 1.5% BRBC were added/well, the plate was shaken at 0.5 RPM for 30 min at 4°C, and the presence or absence of agglutination recorded immediately after. Serum samples showing agglutination inhibition at 1:16,384 were assayed again using higher dilutions. Each sample was tested in duplicate and the HAI titer was expressed as the average of the reciprocal of the highest serum dilution that completely inhibited MRHA.

### Cytokine evaluation

Cytokine levels were measured in culture supernatants of spleen cells harvested two weeks after the last immunization (d42) (5x10^5^ in 200 μl). Cells were cultured in complete RPMI (10% FCS) for 72 h with medium alone (negative control), 5 μg/ml of ConA (Sigma-Aldrich) (positive control), or 10 μg/ml of CfaE or LTB, using a custom-made Bio-Plex® kit for quantification of IFN-gamma, IL-2, IL-4, IL-5, and IL-17 (Bio-Rad, Hercules, CA), following the manufacturer directions.

### Statistical and graphical analyses

Comparisons of peak induration were compared by Mann-Whitney. Serum antibody and HAI titers were normalized by a log_10_ transformation for statistical analysis and compared by t-test. No statistical comparisons were performed on cytokine levels as assays were performed with pooled splenocytes. All statistical analyses were performed using GraphPad Prism Version 6.00 for Mac OS X (Graphpad Software, La Jolla, CA), and a *P* value of <0.05 was considered significant.

## Results

### Evaluation of skin reactogenicity

**Fig 2:**
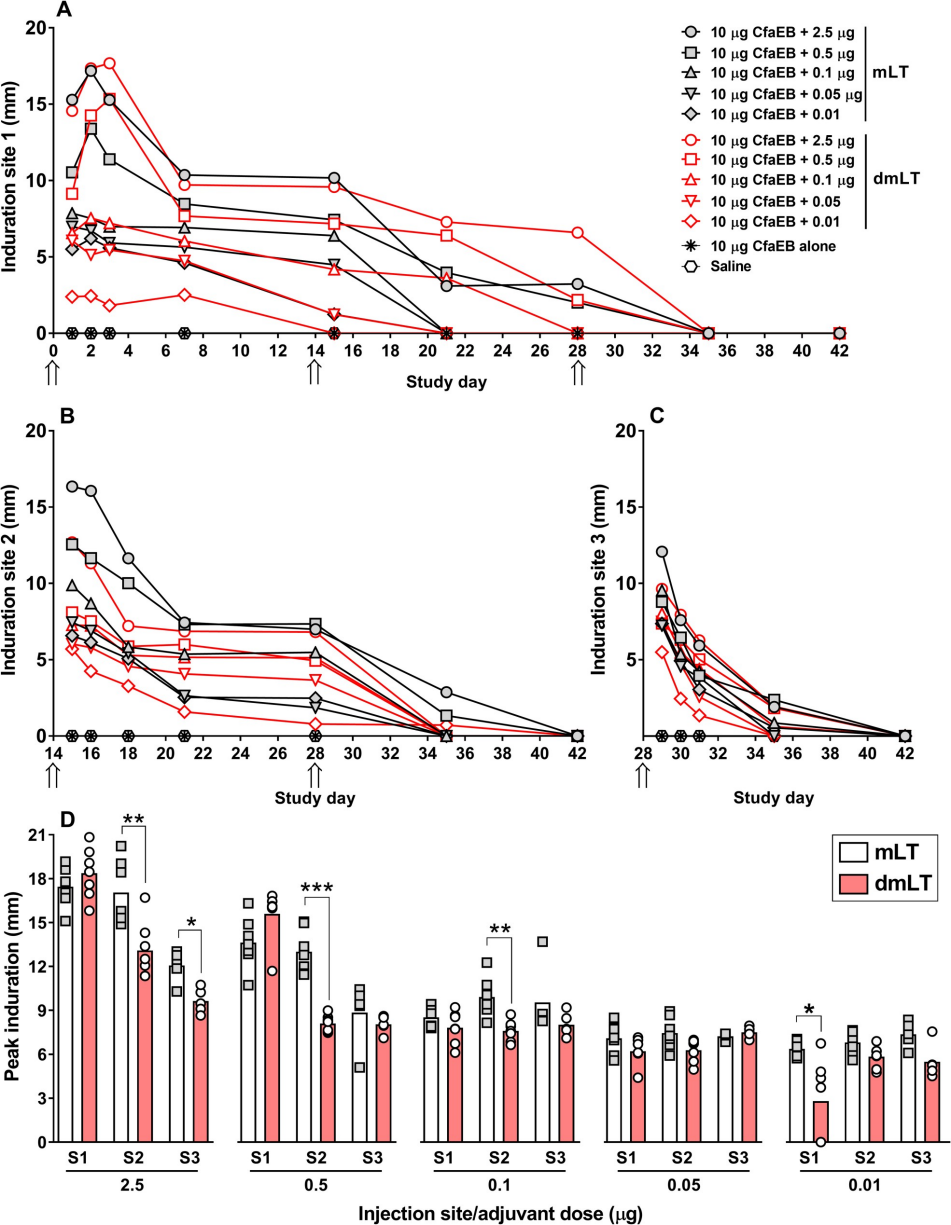
Skin induration. Induration was defined as the mean of the length and width in millimeters, measure on days 1, 2, 3, and every 7 days after each injection until resolution or the end of the study. **(A-C)** Induration measurements at S1, S2, and S3, respectively. The first (d0), second (d14) and third (d28) doses were administered at S1, S2, and S3, respectively. Data is shown as the kinetics of mean induration/group (n = 5-7/group/dose). Arrows indicate days of immunization. **(D)** Individual peak induration measured from each site. Grey squares and white circles represent individual measurements of mice injected with mLT and dmLT, respectively, while bars represent the mean of each group. *P<0.05.

Skin reactogenicity was assessed by three parameters: induration, erythema, and edema at the injection site. For the first two immunizations (d0 and d14), on S1 and S2, we observed a clear increase in induration corresponding to increasing doses of mLT or dmLT (Fig 2A and 2B). At S1, the peak of induration for the two highest doses (i.e. 2.5 and 0.5 μg) of mLT and dmLT occurred at 48 h (d2) and 72 h (d3), respectively, possibly indicating a delay of the response after the first injection of dmLT in comparison to mLT (Fig 2A). In addition, the peak of induration elicited by 0.01 μg of dmLT was significantly lower compared to the same dose of mLT (*P*<0.05; Fig 2D). At S2, the highest measurements of induration were observed earlier, 24 h after the injection (d1) of mLT or dmLT, regardless of the dose (Fig 2B). A statistically significant decrease in the peak of induration was observed with the doses of 2.5, 0.5, and 0.1 μg of dmLT compared to the same doses of mLT (*P*<0.01, *P*<0.001 and *P*<0.01, respectively; Fig 2D). The third immunization (S3) progressed with a less distinct dose-response among the groups, an overall lower degree of induration compared to S1 and S2, and 2.5 μg dmLT promoted significantly less induration then 2.5 μg mLT (*P*<0.05; Fig 2C). Throughout the study, no induration was observed after immunizations with CfaEB alone or saline.

Measurements of erythema and edema did not allow a clear differentiation of the skin reactogenicity induced by mLT or dmLT (S2 Fig). However, Draize score results do suggest a dose-response similar to that observed with the induration promoted by both adjuvants. For example, the highest doses of 2.5 and 0.5 μg of mLT or dmLT at S3 induced the highest levels of erythema and edema, while lower doses of 0.1, 0.05, or 0.01 μg induced gradually lower scores (S2C and S2F Fig). No erythema or edema were observed with the administration of CfaEB alone or saline.

### Skin pathology

**Fig 3:**
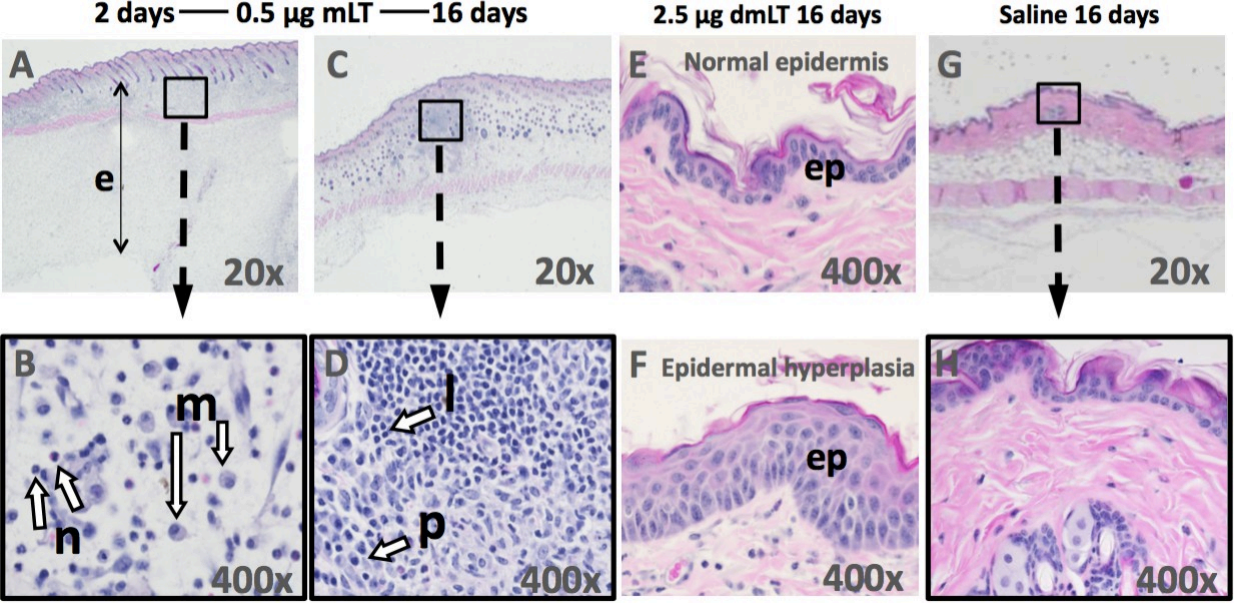
Hematoxylin and eosin-stained skin biopsy specimens obtained following ID injection with mLT or dmLT. Samples were taken sixteen days following the first immunization (S1; C, D, E, F, G, H) and two days following the second immunization (S2; A, B) from animals immunized with 0.5 μg mLT **(**A-D**)**, 2.5 μg dmLT **(**E-F**)** or saline **(**G-H**)**. Images were selected to represent the findings. The pathology of S2 (A and B) was characteristic of acute dermatitis while S1 (C and D) demonstrated chronic dermatitis. Magnification 20x (A, C, and G) and 400x (B, D, E, F, and H). e = edema; n = neutrophil; m = macrophage; p = plasma cell; l = lymphocyte; ep = epidermis.

Histopathology of skin sections from S1 and S2 collected on d16 revealed a biphasic response. Skin from S2, collected 2 days after administration of adjuvant, indicated acute dermatitis that often extended into the subjacent tissue (panniculitis) characterized by a cellular infiltrate composed of neutrophils and macrophages (Fig 3A and 3B), with variable degrees of edema (median score for mLT = 4, dmLT = 3.5, S3B Fig). In mice given mLT, marked dermatitis was present in S2 at the doses of 2.5, 0.5, 0.1, and 0.05 μg and ranged from moderate to marked with 0.01 μg dose (S3A Fig). Mice given dmLT demonstrated moderate to marked dermatitis at the doses of 2.5, 0.5, and 0.1 μg, but mild to moderate dermatitis in mice given 0.05 or 0.01 μg (S3A Fig).

Skin from S1, collected 16 days after administration of the adjuvants, showed a shift to a less severe chronic dermatitis characterized by the presence of plasma cells, lymphocytes and macrophages (Fig 3C and 3D), with considerably less edema (median score for mLT or dmLT = 1, S3B Fig). Mild to moderate dermatitis was present at the doses of 2.5, 0.5, 0.1, and 0.05 μg, but was minimal at the dose 0.01 μg in mice given mLT (S3A Fig). In mice given dmLT, mild to moderate dermatitis was present at the doses of 2.5, 0.5, and 0.1 μg, but was minimal at the doses of 0.05 and 0.01 μg (S3A Fig). No histological changes were observed with the administration of CfaEB or saline. Epidermal hyperplasia was observed in several samples (Fig 3E and 3F), regardless the dose or product compared to skin from saline control mice (Fig 3G and 3H).

Histopathology of skin collected on d42 suggests a trend toward resolution of inflammation (S3C Fig). Skin from S1 and S2, 42 and 28 days after intradermal administration, respectively, showed minimal or no pathology, while samples from S3, 14 days after injection, showed mild chronic dermatitis. Edema was not present in the samples from this time point. Also, there were no differences in skin pathology or severity between mice that received either dose of mLT or dmLT at this time point (S3C Fig).

Overall, S1, S2 and S3 samples obtained at the time of euthanasia indicated an acute dermatitis with edema that, over time, transition to chronic dermatitis prior to resolution by 28 days after injection.

### Adjuvanticity

**Fig 4:**
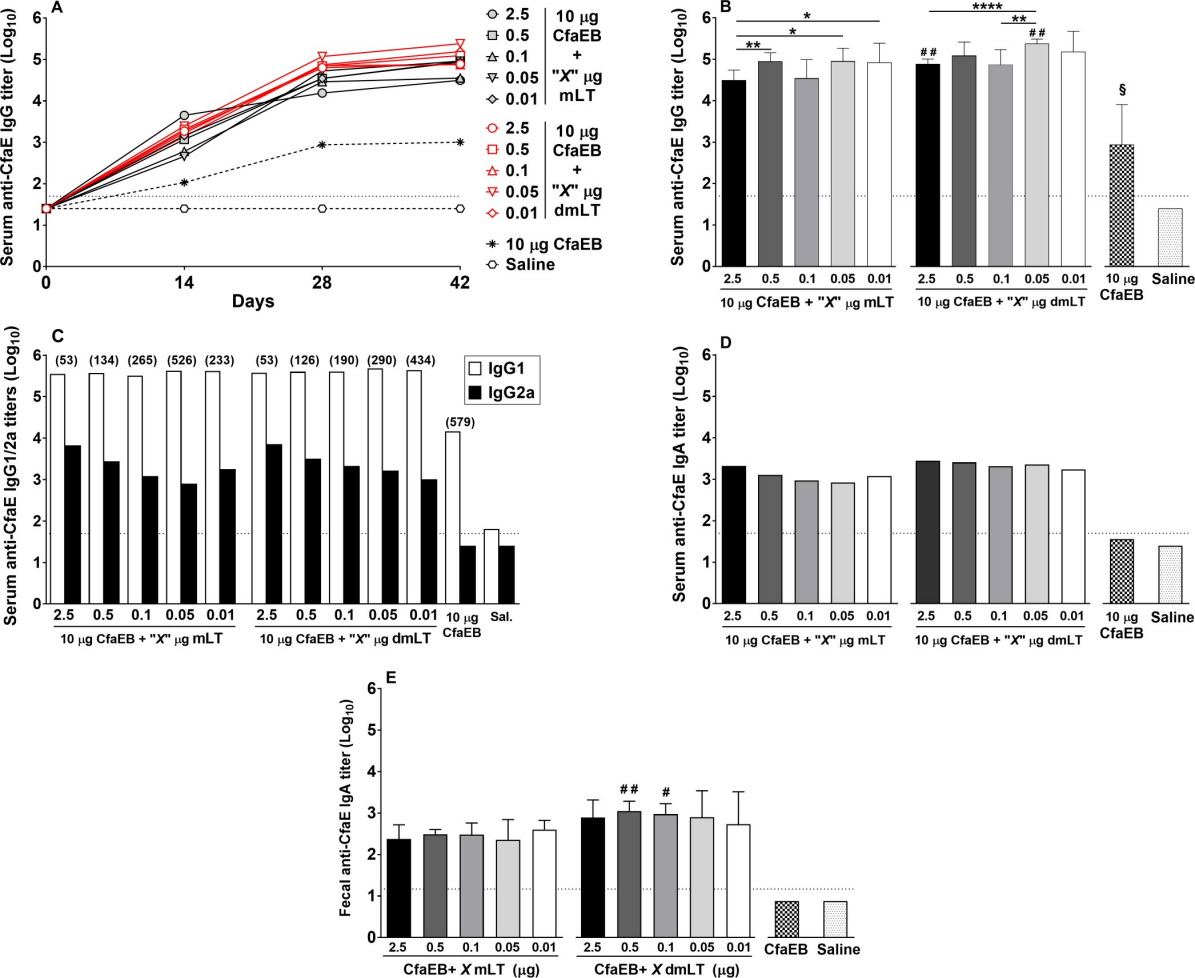
Anti-CfaE antibody response. Mice were immunized by the ID route with varying doses of either mLT or dmLT in the presence of 10 μg CfaEB (see Materials and methods for details). **(A)** Kinetics of anti-CfaE IgG Ab titers for all groups on d0 (evaluated with pool of 7 animals/group), d14 (evaluated with pool of 7 animals/group), d28 (evaluated with pool of 5 animals/group), and d42 (evaluated in individual sera, 5 animals/group) (for clarity, only mean is indicated); **(B)** Geometric mean (+95% confidence interval) titers of log_10_ transformed anti-CfaE IgG Ab titers on d42 for all groups; **(C)** Serum anti-CfaE IgG1 and IgG2a Ab titers on d42 performed with pooled sera (bars represent the mean of duplicate assays); IgG1/IgG2a ratios are shown in parentheses; **(D)** Serum anti-CfaE Ab IgA antibody titers on d42 performed with pooled sera (bars represent the mean of duplicate assays); (E) Fecal IgA anti-CfaE Ab titers on d35. Dotted horizontal line represents the limit of detection for the assays. *P<0.05, **P<0.01, and ***P<0.001 indicated for statistical comparisons performed within mLT or dmLT groups; ^**#**^*P*<0.05, ^**# #**^*P*<0.01, and ^**# # #**^*P*<0.001 indicated for statistical comparisons performed between the same dose of mLT and dmLT.

High levels of serum anti-CfaE IgG Ab were observed after the first immunization (d14) with all doses of mLT or dmLT. The second immunization led to an increase of those levels, which plateaued by d28 (Fig 4A). On d42, after completion of the immunization schedule, anti-CfaE IgG Ab levels titers elicited by the immunization with 2.5 μg of mLT + CfaEB (mean log_10_ titer: 31,095) were significantly lower than those from groups immunized with 0.5 (88,682; *P*<0.01), 0.05 (89,790; *P*<0.05) or 0.01 μg mLT (83,907; *P*<0.05) (Fig 4B). Among the groups immunized with dmLT, anti-CfaE IgG Ab titers were significantly higher in the group immunized with CfaEB + 0.05 μg of dmLT (239,590) in comparison to groups that received either 0.1 μg (73,321; *P*<0.01) or 2.5 μg dmLT (77,140; *P*<0.0001) (Fig 4B). When comparing mLT to dmLT, the doses of 2.5 and 0.05 μg of dmLT elicited levels of anti-CfaE IgG Abs that were significantly higher when compared to the same doses of mLT (*P*<0.01 for both comparisons). Finally, all doses of either adjuvant produced levels of anti-CfaE IgG Abs that were significantly higher than immunization with CfaEB alone.

Immunization with dmLT with doses ranging from 2.5 to 0.05 μg promoted a more balanced Th1/Th2 response, compared to the same doses of mLT, as judged by overall lower anti-CfaE IgG1:IgG2a ratios (Fig 4C). Although CfaEB given alone failed to elicit detectable levels of anti-CfaE IgA Abs, the co-administration with both adjuvants, mLT and dmLT, at any of the doses evaluated, led to similar levels (Fig 4D). However, significantly higher fecal anti-CfaE IgA levels were observed from groups immunized with 0.5 and 0.1 μg dmLT compared to the same doses of mLT (*P*<0.01 and *P*<0.05, respectively; Fig 4E)

**Fig 5:**
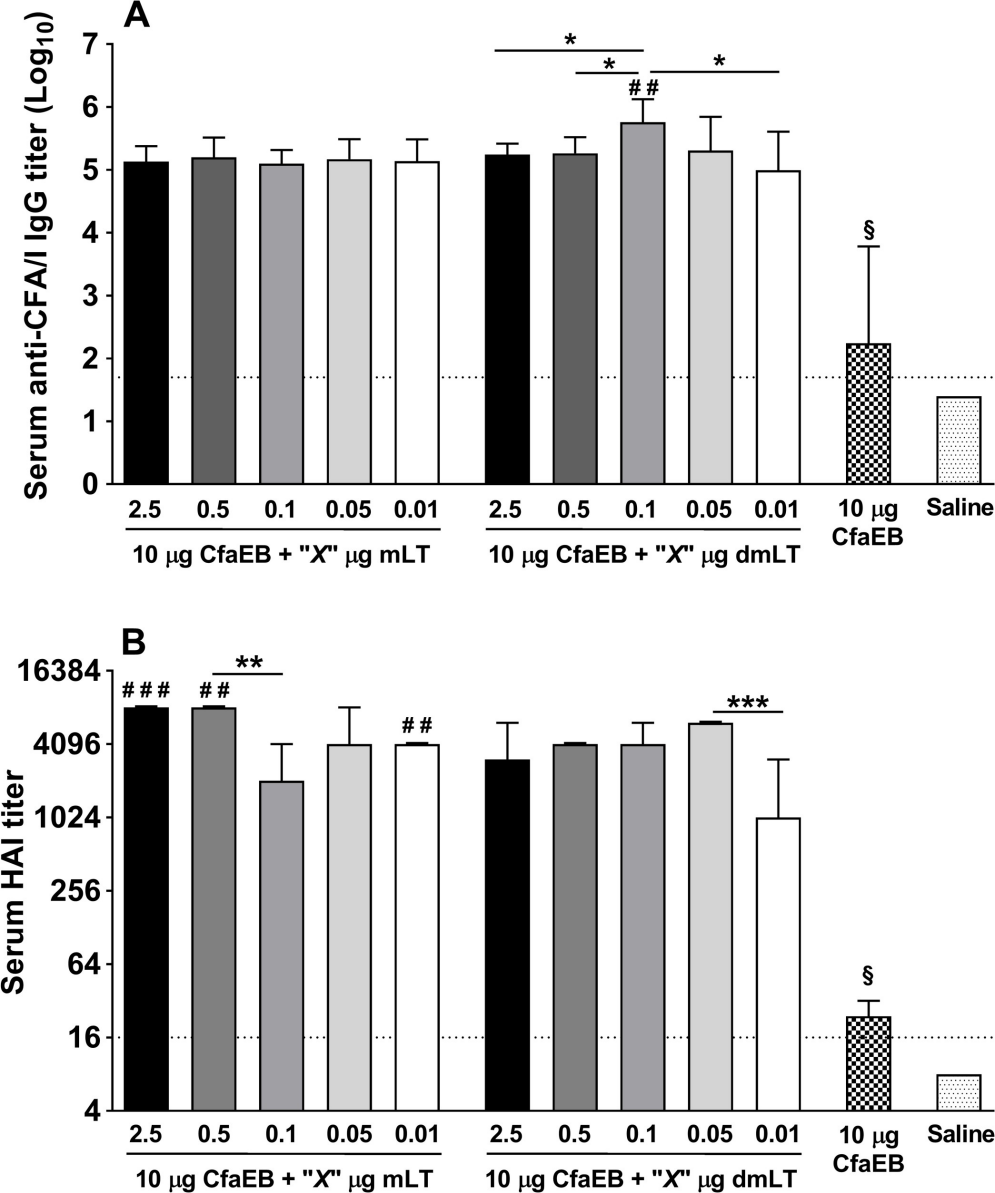
Evaluation of anti-CFA/I IgG and functional neutralizing antibodies against CFA/I^+^ ETEC (strain H10407). Data are from d42 sera. **(A)** Geometric mean titers (+95% confidence interval) of log_10_ transformed of anti-CFA/I IgG Ab titers on d42 for all groups (n = 5/group); **(B)** Functional neutralizing antibodies against CFA/I^+^ ETEC strain H10407; bars represent the median ± ranges for each group (n = 5/group). Dotted horizontal line represents the limit of detection for the assays. *P<0.05, **P<0.01, and ***P<0.001 indicated for statistical comparisons performed within mLT or dmLT groups; ^**#**^*P*<0.05, ^**# #**^*P*<0.01, and ^**# # #**^*P*<0.001 indicated for statistical comparisons performed between the same dose of mLT and dmLT.

We also tested the response elicited by CfaEB against the whole fimbriae, CFA/I. The levels of anti-CFA/I IgG Abs were similar among all groups that receive mLT, regardless the immunization dose (Fig 5A). Immunization with dmLT also elicited similar anti-CFA/I IgG levels, except for the dose of 0.1 μg, which promoted levels significantly higher in comparison to the same dose of mLT (*P*<0.01), as well as higher than dmLT at 2.5, 0.5 and 0.01 μg (*P*<0.05 for all comparisons; Fig 5A). In addition to the direct measurement of serum Ag-specific IgG and IgA Abs levels by ELISA, we employed the hemagglutination inhibition (HAI) assay to assess the levels of functional Abs blocking RBC agglutination driven by an ETEC strain expressing CFA/I CF, i.e. H10407. All doses of mLT and dmLT were able to elicit very high levels of anti-CFA/I functional Abs (Fig 5B). Immunization with CfaEB plus 2.5, 0.5 or 0.01 μg of mLT promoted anti-CFA/I HAI titers that were significantly higher in comparison to the same doses of dmLT (median 8,192 vs 3,072, 8,192 vs 4096, and 4,096 vs 1,024, respectively; Fig 5B). CfaEB alone elicited only modest HAI titers, which were significantly lower when compared to any dose of either adjuvant (*P*<0.0001, Fig 5B).

### Immunogenicity

**Fig 6:**
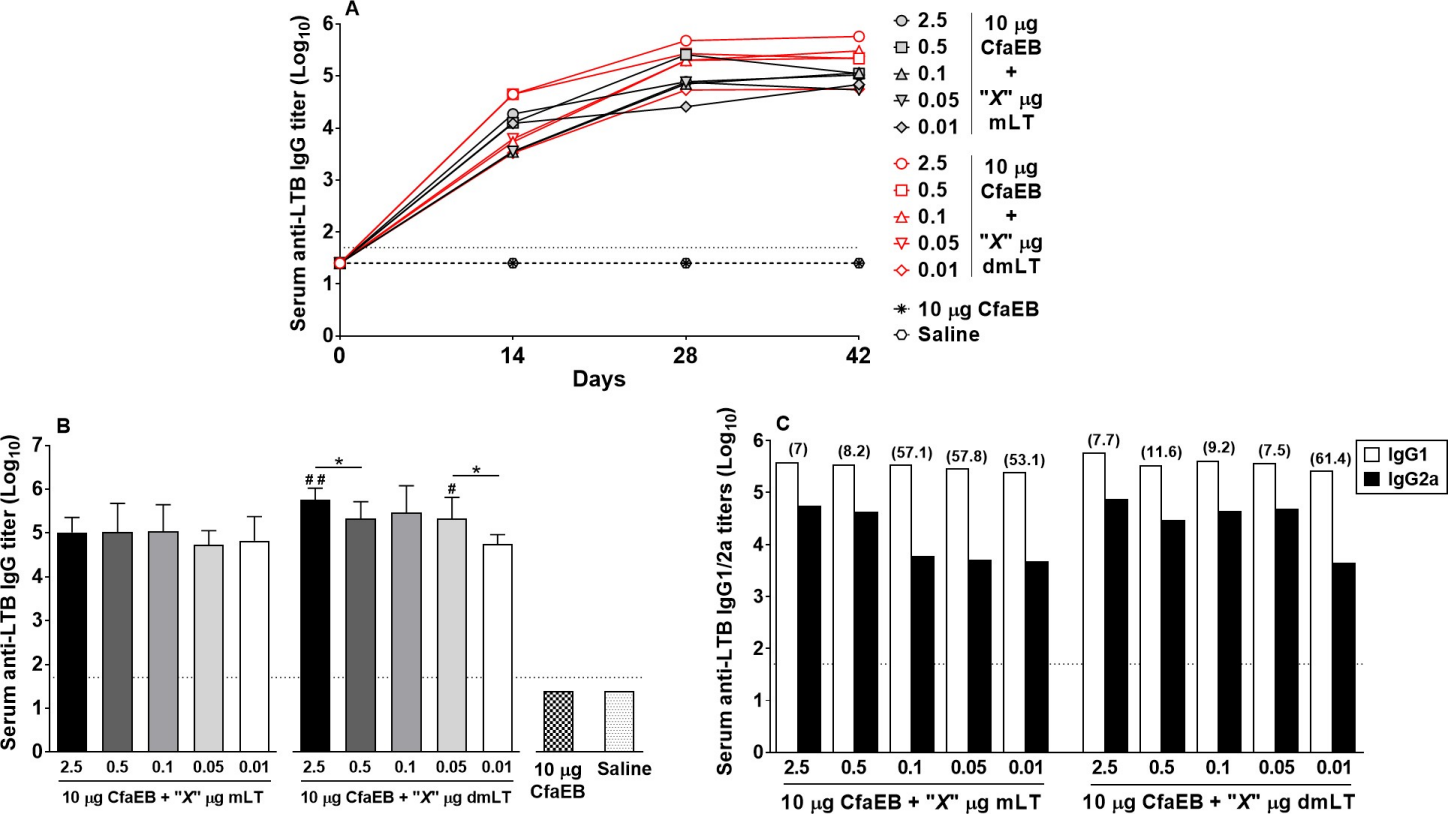
Serum anti-LTB IgG titers. **(A)** Kinetics of anti-LTB IgG Ab titers on d0 (evaluated with pool of 7 animals/group), d14 (evaluated with pool of 7 animals/group), d28 (evaluated with pool of 7 animals/group), and d42 (evaluated in individual sera, 5 animals/group); **(B)** Mean geometric log_10_ transformed anti-LTB Ab IgG titers on d42 for all groups; **(C)** Anti-LTB IgG1 and IgG2a Ab titers on d42 performed with pooled sera (bars represent the mean of duplicate assays); IgG1/IgG2a ratios are shown in parentheses. Dotted horizontal line represents the limit of detection for the assay. *P<0.05, **P<0.01, and ***P<0.001 indicated for statistical comparisons performed within mLT or dmLT groups; ^**#**^*P*<0.05, ^**# #**^*P*<0.01, and ^**# # #**^*P*<0.001 indicated for statistical comparisons performed between the same dose of mLT and dmLT.

Since both adjuvants, mLT and dmLT, are also antigenic, we evaluated the serum IgG response against the B subunit of the LT toxin (LTB), as a measure of antigenicity. High levels of serum anti-LTB IgG Abs were observed after the first immunization (d14) with all doses of mLT or dmLT (Fig 6A). The second immunization led to an increase of those levels (d28), with no further increase by d42. Here, after the final immunization, we observed that while all doses of mLT led to similar levels of anti-LTB IgG, dmLT, used at 2.5 and 0.05 μg, elicited significantly higher Ag-specific IgG titers, compared to the same doses of mLT (*P*<0.05 for both comparisons, Fig 6B). In addition, among the groups that received dmLT, significant higher anti-LTB IgG levels were observed with 2.5 compared to 0.5 μg (*P*<0.05), and 0.05 compared to 0.01 μg (*P*<0.05) (Fig 6B), suggesting a dose-response.

The evaluation of anti-LTB IgG1 and IgG2a subclasses revealed a more balanced response among the groups that received dmLT between the doses of 2.5 to 0.05 μg in comparison to the ratios observed with the same doses of mLT, in a fashion similar to the results observed with anti-CfaE IgG subclasses (Fig 6C).

### Cytokine response

**Fig 7:**
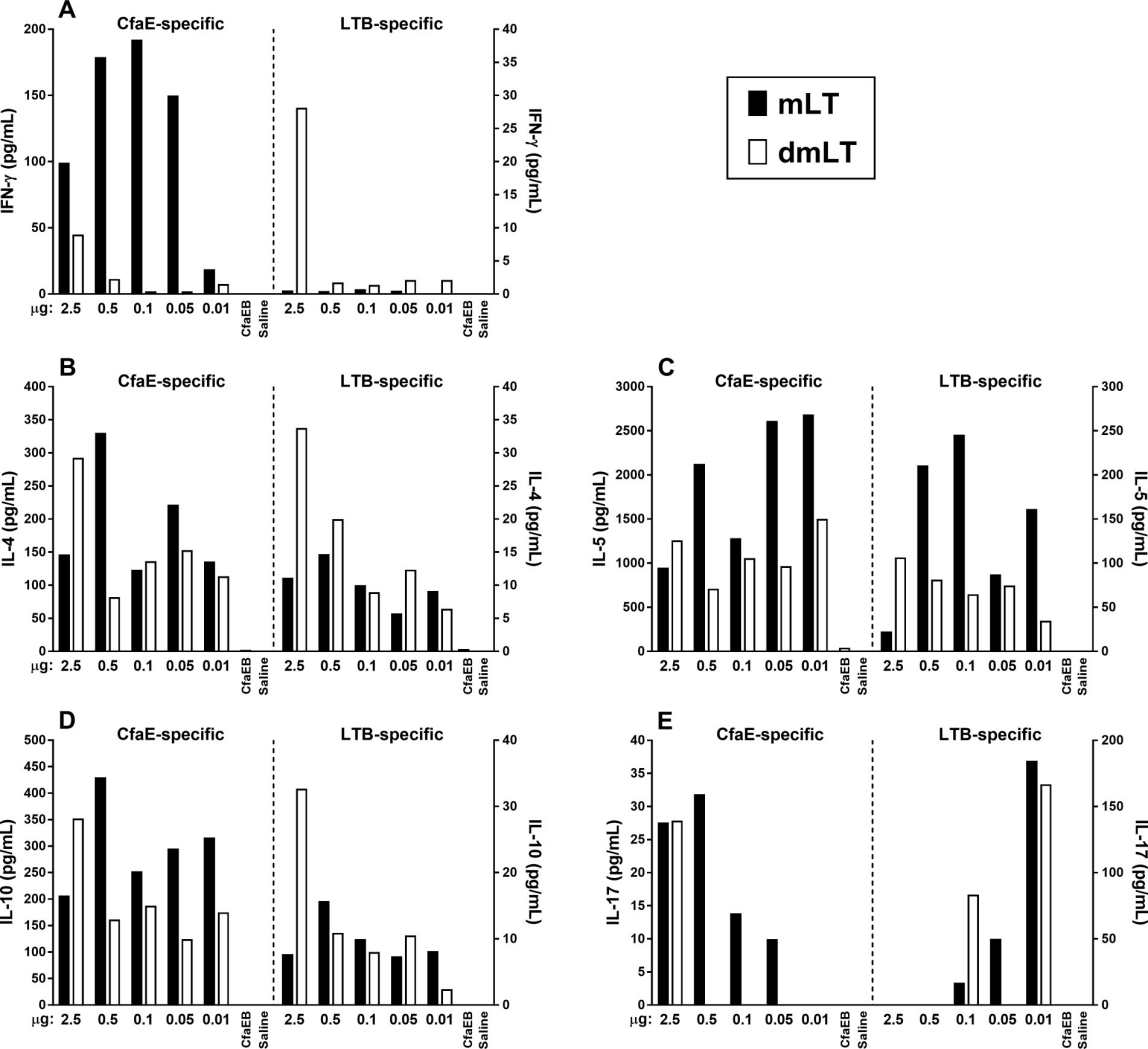
Evaluation of cytokine secretion. Two weeks after the third immunization, animals were euthanized and three spleens from each group were homogenized, pooled and cultured as described in material and methods section. Supernatants were assayed in duplicate by Luminex and the results are shown as net values after subtractions of the cytokine levels obtained with medium only. All samples responded with very high levels of all cytokines under ConA stimulation. Bars represent the average result of duplicate assays. **(A)** Interferon-gamma; **(B)** IL-4; **(C)** IL-5; **(D)** IL-10; **(E)** IL-17.

All cytokines, except IL-17, (i.e. IFN-γ, IL-4, IL-5, IL-10) were produced in larger quantities under *in vitro* stimulation with CfaE than with LTB, and for all cytokines considered, negligible levels were observed in the two control groups immunized with either CfaEB alone or saline (Fig 7). For IFN-γ, samples from animals immunized with CfaEB plus the various doses of mLT secreted higher amounts of the cytokine under CfaE stimulation in comparison to the levels produced by mice that received corresponding doses of CfaEB plus dmLT (Fig 7A). Interestingly, IFN-γ levels produced under LTB stimulation were overall higher with samples from the groups immunized with CfaEB plus dmLT, markedly with 2.5 μg (Fig 7A).

Immunization with mLT or dmLT prompted secretion of IL-4 in response to the stimulation with CfaE, with no clear association to adjuvant dose (Fig 7B). Although the response to *in vitro* stimulation with LTB led to IL-4 levels lower than those induced by CfaE, the results suggest a dose-response effect, which was more pronounced for groups immunized with increasing doses of dmLT (6.4, 12.3, 9, 20, and 34 pg/mL for 0.01, 0.05, 0.1, 0.5, and 2.5 μg of dmLT, respectively) (Fig 7B).

No clear dose-response was observed for CfaE-specific IL-5 secretion over the various doses of either adjuvant, with the highest secretion levels detected at the lowest doses of 0.01 μg of mLT (2,684 pg/mL) or dmLT (1,495 pg/mL) (Fig 7C). An LTB-specific IL-5 dose-response was observed in the groups immunized with increasing doses of dmLT (35, 75, 65, 81, and 106 pg/mL for 0.01, 0.05, 0.1, 0.5, and 2.5 μg of dmLT, respectively), although the highest levels were observed in groups immunized with mLT (e.g. 245.4 pg/mL with 0.1 μg mLT) (Fig 7C).

Except at the highest dose (2.5 μg), mLT led to secretion of higher CfaE-specific IL-10 levels compared to dmLT (Fig 7D). A dose-response was seen in the groups immunized with increasing doses of dmLT under *in vitro* stimulation with LTB (2.4, 10.5, 8, 11, and 32 pg/mL for 0.01, 0.05, 0.1, 0.5, and 2.5 μg of dmLT, respectively) (Fig 7D).

The quantification of IL-17 showed a very distinct pattern in comparison to the other cytokines evaluated (Fig 7E). First, the levels of LTB-specific IL-17 secretion were higher than CfaE-specific IL-17. Second, the groups that received lower doses of mLT or dmLT secreted the highest levels of the cytokine. For instance, 0.01, 0.05 and 0.1 μg mLT led to secretion of 184, 50, and 17 pg/mL of IL-17, respectively, while 0.01 and 0.1 μg of dmLT led to the secretion of 167 and 84 pg/mL, respectively. Inversely, groups that received higher doses of either adjuvant were more likely to produce IL-17 under *in vitro* stimulation with CfaE. Specifically, IL-17 production was detected in groups that received 2.5, 0.5, 0.1 and 0.05 μg of mLT, or 2.5 μg of dmLT (Fig 7E).

## Discussion

There are no adjuvants approved for ID immunization. The most common adjuvant present in our current vaccines is Aluminum Hydroxide (Al(OH)_3_), which is approved for intramuscular administration. When Al(OH)_3_ was evaluated by the ID route in BALB/c mice, it induced heavy infiltration of inflammatory cells, caused skin ulceration, and formation of abscesses filled with a white/silver depot that did not resolve for months [31]. More recently, Oreskovic and cols. also observed large deposition of neutrophils, signs of necrosis and occasional generation of abscesses after ID use of Al(OH)_3_ as adjuvant for KLH in pigs [32]. Altogether, those results do not support the use of Al(OH)_3_ for ID immunization. GLA-AF, an emulsion-free aqueous formulation with glucopyranosyl lipid adjuvant, a TLR-4 agonist, has been evaluated for ID immunization with an H5-VLP influenza vaccine and showed low skin reactogenicity in guinea pigs and stimulated strong anti-H5 responses in C57BL/6 mice [33].

We have previously used mLT as an oral and intranasal adjuvant to investigate the immunogenicity of CfaE in mice and *Aotus nancymaae* monkeys [5]. We have also administered mLT by the ID route in screening CS6 CF vaccine candidates in mice [13]. However, the choice of adjuvant is not always obvious and must be guided by characteristics of the formulation, route of administration, quality of the desired immune response, and safety profile. Particularly relevant to ETEC vaccines, the use of attenuated versions of LT as adjuvants, such as mLT or dmLT, can also elicit anti-toxin responses, which can confer some protection against LT^+^ only ETEC [11]. In comparison to wild-type LT, both adjuvants exhibit attenuated toxicity profiles judged by their reduced ability to induce *in vitro* cAMP (1,000-fold lower compared to LT) and reduced potential to promote intestinal liquid accumulation when orally administered to mice [4].

Dermal immunization routes can accentuate the observation of local reactogenicity, which otherwise may not be easily measurable as for example in subcutaneous or intramuscular immunizations. Here we observed that both adjuvants, mLT and dmLT, were locally reactogenic when injected ID, in a dose-dependent fashion. However, the results suggest that dmLT tends to be less reactogenic than mLT, judged by the reduced induration seen with some of the doses and by the slower peak of response observed after the first dose. This may be explained by the reduced toxicity of dmLT, which possesses an additional amino acid substitution (L211A). Two key observations regarding local reactogenicity were that (1) skin reactogenicity (induration, edema and erythema) tended to spontaneously resolve and (2) responses were smaller and of shorter duration with each subsequent immunization.

The mechanism of action of dmLT (and other LT-derived adjuvants) has recently been reviewed by Clements and Norton [34]. The activation of epithelial cells at the site of immunization promotes secretion of IL-8 and G-CSF and consequent activation of the innate immune system, with recruitment of dendritic cells (DCs) and upregulation of co-stimulatory molecules, such as CD80 and CD86 [34]. Once activated and loaded with the antigen, DCs can migrate to draining lymph nodes and present the antigen to T helper cells to support B cell activation and differentiation. Although our approach did not specifically characterize cell subpopulations at the immunization sites, the results resemble the findings of Heine *et al*., in a study using dmLT as an adjuvant for the intradermal delivery of *Shigella* subunit vaccine candidates [35]. As with that study, our results point to a biphasic process, where neutrophils and macrophages accumulate soon after the intradermal injections with mLT or dmLT, followed later by cells of the adaptive immune system, such as T and B cells. Interestingly, the degree of cell accumulation at the immunization site seems to have only a modest correlation to the immune response elicited, as we recently demonstrated [36]. Those observations suggest that even the lowest dose of mLT or dmLT (0.01 μg) can lead to activation of DCs, regardless of the size of cell infiltration at the immunization site. The current study did not address whether the antigen (i.e. CfaEB) can modulate the reactogenicity induced by the adjuvants. However, we have not observed differences in the induration response in mice immunized with 0.1 μg dmLT +/- 10 μg CfaEB (unpublished observation), although results could vary depending on the dose of each component.

Recombinant mLT and dmLT functioned as potent adjuvants in this study. Even at the lowest dose (0.01 μg), immunization with either adjuvant plus CfaEB elicited significantly higher levels of anti-CfaE IgG and anti-CFA/I functional HAI blocking Abs, compared to the protein alone. Interestingly, in the case of serum anti-CfaE IgG responses, lower doses of either adjuvant seemed to favor higher antibody titers, while an opposite trend was observed with the functional response. It is possible that the higher levels of anti-CfaE IgG2a Abs observed with the higher doses of adjuvant contributed to higher functional responses. Although anti-CfaE IgG1/IgG2a ratios were dose-dependent for both adjuvants, dmLT promoted a more balanced response between the doses of 2.5 and 0.05 μg. Immunization with CfaEB was able to promote high levels of anti-CFA/I antibodies, which indicates that both minor and major subunits in the fusion protein were antigenic.

High serum levels of anti-toxin IgG Abs (evidenced by the response against the immunodominant B subunit of LT, i.e. LTB) were observed with both adjuvants at even the lowest dose level (0.01 μg). For two of the adjuvant doses evaluated, 2.5 and 0.05 μg, dmLT promoted higher anti-toxin IgG levels in comparison to mLT. Although we have not evaluated anti-toxin functional responses, we and others have demonstrated that immunization with dmLT can generate LT-neutralizing responses [37, 38]. We also documented anti-LT neutralizing responses after intradermal immunization of volunteers with mLT in Phase 1 and Phase 2b clinical trials (clinicaltrials.gov identifier NCT01644565 and NCT01922856) [39, 40]. Interestingly, detectable anti-LTB IgA Ab titers (107.0), albeit low, were only observed in the group immunized with 2.5 μg dmLT. Fecal anti-toxin IgA Abs were not observed. It is possible that, while mLT and dmLT retain antigenicity and elicit very high IgG titers, IgA responses might demand high doses of the protein.

The plateau in anti-CfaE and LTB IgG Ab titers observed after the second immunization suggests that a reduced schedule, with prime and single intradermal boost, could generate similar or even higher antigen-specific responses. Further studies to refine the immunization schedule and investigate the impact of the number of and time between immunizations on the immune response would be informative.

LT-derived adjuvants can promote the secretion of Th1, Th2, and Th17 cytokines [34, 41]. However, no comparison between mLT and dmLT has been performed to define whether similar mechanisms are elicited by both proteins. Regardless of dose, ID immunization with mLT leads to a more vigorous IFN-γ response against the co-administered antigen (i.e. CfaEB), while only marginal responses were detected against LTB. Interestingly, the levels of anti-CfaE IgG2a elicited by mLT were lower in comparison to dmLT, given the high IFN-γ response seen. It is possible that the counter regulatory production of CfaE-specific IL-10 contributed to this result. It seems reasonable, though, to speculate that the stronger stimulation of IFN-γ production is partially responsible for the increased skin reactogenicity observed with mLT.

The pattern of response of IL-4, IL-5 and IL-10 was more sensitive to the amount of adjuvant, particularly dmLT. For instance, *in vitro* stimulation with LTB led to dose- response effects, while no clear dose-response correlation was observed with *in vitro* stimulation with CfaE. These observations suggest that dmLT can promote more gradual responses than mLT, which can be important from a clinical standpoint in allowing better dose-effect control. However, the lowest dose of either adjuvant was sufficient to prime responses against the co-administered antigen. These observations have important implications for vaccine development and highlight the need for properly choosing the antigen and adjuvant doses. Further investigations should focus on the antigen:adjuvant ratio.

As other authors have reported, we also observed that dmLT activates IL-17 [41, 42]. Herein we observed that mLT can also promote IL-17 responses. Curiously, IL-17 secretion upon *in vitro* recall with LTB was more likely when lower doses of adjuvants were used for the immunizations, while higher doses favored IL-17 production under CfaE stimulation. For the latter, it seems that the immunization with mLT was more efficient in priming Th17 responses to CfaE at doses between 2.5 and 0.05 μg, while dmLT only primed Th17 responses at the highest dose of 2.5 μg, possibly due to its higher degree of detoxification. Since IL-17 can promote IgA [42], IgG2a production [43], and support inflammatory processes [44], it is important to find the right balance between those responses.

In the recent past, our group has evaluated mLT for transcutaneous (clinicaltrial.gov identifier NCT01382095) and intradermal vaccination (NCT01644565) (manuscripts in preparation) [16]. Our results give strong support to the use of mLT and dmLT as intradermal adjuvants. Although dose-dependent local reactogenicity was observed with either adjuvant, it tends to resolve over time. Between them, mLT seems to be more inflammatory and elicit stronger IFN-γ responses. Even minute amounts of either adjuvant (i.e. 0.01 μg) were able to elicit specific IgG and IgA, as well as functional responses against the co-administered antigen. In addition, mLT and dmLT were very antigenic and elicited a systemic anti-toxin response, an advantageous characteristic for ETEC vaccines. Altogether, our results demonstrate the importance of carefully evaluating the dose of adjuvant and the ratio of protein:adjuvant, and should further aid the development of mLT or dmLT as adjuvants for parenteral vaccine formulations.

## Supporting information

S1 File Raw data for Figs 2–7 and S2 and S3 Figs. (PDF)

S1 Fig Dose verification. **(A)** Western blot analysis of mLT standards (0.0025, 0.005, 0.025, and 0.05 μg) and dose formulations for Study Groups 1–12 using rabbit polyclonal sera generated against the B subunit of LT. The expected amounts of mLT/dmLT for the analysis based on the original concentration of each group’s formulation are as follows: #1–0.025 μg (100-fold dilution of the original 2.5 μg of mLT); #2–0.025 μg (20-fold dilution of the original 0.5 μg of mLT); #3–0.05 μg (2-fold dilution of the original 0.1 μg of mLT); #4–0.025 μg (2-fold dilution of the original 0.05 μg of mLT); #5–0.005 μg (2-fold dilution of the original 0.01 μg of mLT); #6–0.025 μg (100-fold dilution of the original 2.5 μg of dmLT); #7–0.025 μg (20-fold dilution of the original 0.5 μg of dmLT); #8–0.05 μg (2-fold dilution of the original 0.1 μg of dmLT); #9–0.025 μg (2-fold dilution of the original 0.05 μg of dmLT); #10–0.005 μg (2-fold dilution of the original 0.01 μg of dmLT); #11–10 μg CfaEB alone (2-fold dilution); and #12- Saline (2-fold dilution). **(B)** SDS page gel analysis of CfaEB standards (0.1, 0.5, 1.0, and 1.5 μg) and dose formulations for Study Groups 1–12.
(TIF)

S2 Fig Adapted Draize scores of erythema and edema. Mice were immunized with dscCfaEB and varing doses of mLT or dmLT by the ID route on days 0, 14, and 28 at sites 1, 2 and 3, respectively. Based on Adapted Draize scores (Table 2), erythema and edema at the injection sites were observed and recorded 24, 48 and 72 hours after each immunization as well as every 7 days until resolution or end of the study. Data is presented as median peak of erythema or edema ± range. (**A-B-C)** Erythema; (**D-E-F)** Edema.
(TIF)

S3 Fig Skin pathology scores. Mice were immunized with dscCfaEB and varying doses of mLT or dmLT by the ID route on days 0, 14, and 28. On day 16 of the immunization protocol, two animals from each group were euthanized and skin samples from the first (S1) and second (S2) immunizations were collected, corresponding to 16 and 2 days after each site immunization, respectively. On day 42, skin samples were collected from sites 1, 2, and 3 (S3) from two more animals, which corresponded to 42, 28, and 14 days after each site immunization, respectively. Samples were preserved and stain by hematoxylin and eosin for histopathology evaluation. The presence of edema and pathology were scored as described in the material and methods section. **(A)** Skin pathology scores for S1 and S2 collected on day 16. **(B)** Edema scores for S1 and S2 collected on day 16. **(C)** Skin pathology scores for S1, S2 and S3 collected on day 42. Bars represent the average score while individual values are shown as squares for mice immunized with mLT or circles for mice immunized with dmLT.
(TIF)

S4 Fig Raw images—Dose verification. (PDF)

## References

[1] Mutsch M, Zhou W, Rhodes P, Bopp M, Chen RT, Linder T, et al Use of the inactivated intranasal influenza vaccine and the risk of Bell's palsy in Switzerland. N Engl J Med. 2004;350(9):896–903. 10.1056/NEJMoa030595 . 14985487

[2] da Hora VP, Conceicao FR, Dellagostin OA, Doolan DL. Non-toxic derivatives of LT as potent adjuvants. Vaccine. 2011;29(8):1538–44. 10.1016/j.vaccine.2010.11.091 . 21163247

[3] Rappuoli R, Pizza M, Douce G, Dougan G. <LT structure & adjuvanticity_IT_1999pdf.pdf>. Immunology Totay. 1999;20(11):493–500. 10.1016/s0167-5699(99)01523-6 10529776

[4] Norton EB, Lawson LB, Freytag LC, Clements JD. Characterization of a mutant Escherichia coli heat-labile toxin, LT(R192G/L211A), as a safe and effective oral adjuvant. Clinical and vaccine immunology: CVI. 2011;18(4):546–51. 10.1128/CVI.00538-10 21288994 PMC3122563

[5] Sincock SA, Hall ER, Woods CM, O'Dowd A, Poole ST, McVeigh AL, et al Immunogenicity of a prototype enterotoxigenic Escherichia coli adhesin vaccine in mice and nonhuman primates. Vaccine. 2016;34(2):284–91. 10.1016/j.vaccine.2015.11.017 . 26597148

[6] Holmgren J, Bourgeois L, Carlin N, Clements J, Gustafsson B, Lundgren A, et al Development and preclinical evaluation of safety and immunogenicity of an oral ETEC vaccine containing inactivated E. coli bacteria overexpressing colonization factors CFA/I, CS3, CS5 and CS6 combined with a hybrid LT/CT B subunit antigen, administered alone and together with dmLT adjuvant. Vaccine. 2013;31(20):2457–64. 10.1016/j.vaccine.2013.03.027 . 23541621

[7] Lundgren A, Bourgeois L, Carlin N, Clements J, Gustafsson B, Hartford M, et al Safety and immunogenicity of an improved oral inactivated multivalent enterotoxigenic Escherichia coli (ETEC) vaccine administered alone and together with dmLT adjuvant in a double-blind, randomized, placebo-controlled Phase I study. Vaccine. 2014;32(52):7077–84. 10.1016/j.vaccine.2014.10.069 . 25444830

[8] Lapa JA, Sincock SA, Ananthakrishnan M, Porter CK, Cassels FJ, Brinkley C, et al Randomized clinical trial assessing the safety and immunogenicity of oral microencapsulated enterotoxigenic Escherichia coli surface antigen 6 with or without heat-labile enterotoxin with mutation R192G. Clinical and vaccine immunology: CVI. 2008;15(8):1222–8. 10.1128/CVI.00491-07 18579693 PMC2519302

[9] Akhtar M, Chowdhury MI, Bhuiyan TR, Kaim J, Ahmed T, Rafique TA, et al Evaluation of the safety and immunogenicity of the oral inactivated multivalent enterotoxigenic Escherichia coli vaccine ETVAX in Bangladeshi adults in a double-blind, randomized, placebo-controlled Phase I trial using electrochemiluminescence and ELISA assays for immunogenicity analyses. Vaccine. 2018 10.1016/j.vaccine.2018.11.040 . 30473185 PMC6717083

[10] Dickinson BL, Clemens JD. Dissociation of Escherichia coli Heat-Labile Enterotoxin Adjuvanticity from ADP-Ribosyltransferase Activity. Infection and immunity. 1995;63(5):1617–23. 7729864 10.1128/iai.63.5.1617-1623.1995 PMC173200

[11] Behrens RH, Cramer JP, Jelinek T, Shaw H, von Sonnenburg F, Wilbraham D, et al Efficacy and safety of a patch vaccine containing heat-labile toxin from Escherichia coli against travellers' diarrhoea: a phase 3, randomised, double-blind, placebo-controlled field trial in travellers from Europe to Mexico and Guatemala. The Lancet Infectious Diseases. 2014;14(3):197–204. 10.1016/S1473-3099(13)70297-4 24291168

[12] Poole ST, McVeigh AL, Anantha RP, Lee LH, Akay YM, Pontzer EA, et al Donor strand complementation governs intersubunit interaction of fimbriae of the alternate chaperone pathway. Mol Microbiol. 2007;63(5):1372–84. 10.1111/j.1365-2958.2007.05612.x . 17302815

[13] Poole ST, Maciel M Jr., Dinadayala P, Dori KE, McVeigh AL, Liu Y, et al Biochemical and Immunological Evaluation of Recombinant CS6-Derived Subunit Enterotoxigenic Escherichia coli Vaccine Candidates. Infection and immunity. 2019;87(3). 10.1128/IAI.00788-18 . 30602504 PMC6386543

[14] Rollenhagen JE, Jones F, Hall E, Maves R, Nunez G, Espinosa N, et al Establishment, validation and application of a New World Primate model of ETEC disease for vaccine development. Infection and immunity. 2018 10.1128/IAI.00634-18 30510102 PMC6346130

[15] Savarino SJ, McKenzie R, Tribble D, Porter C, O'Dowd A, Cantrell JA, et al Prophylactic Efficacy of Hyperimmune Bovine Colostral Antiadhesin Antibodies Against Enterotoxigenic Escherichia coli Diarrhea: A Randomized, Double-Blind, Placebo-Controlled, Phase 1 Trial. Journal of Infectious Diseases. 2017 10.1093/infdis/jix144 28541500

[16] Harro C, Gutierrez RL, Talaat K, Porter C, Riddle MS, Maciel M, Jr., et al., editors. Protective efficacy of an enterotoxigenic E. coli fimbrial tip adhesin vaccine given with LTR192G by intradermal vaccination against experimental challenge with CFA/I-ETEC in adult volunteers. 50th US-Japan Cooperative Medical Sciences Program Joint Panel Conference on Cholera and Other Bacterial Enteric Infections; 2016; Bethesda, MD. Bethesda, MD2016.

[17] Kim YC, Jarrahian C, Zehrung D, Mitragotri S, Prausnitz MR. Delivery systems for intradermal vaccination. Curr Top Microbiol Immunol. 2012;351:77–112. 10.1007/82_2011_123 21472533 PMC3173582

[18] Nelson KS, Janssen JM, Troy SB, Maldonado Y. Intradermal fractional dose inactivated polio vaccine: a review of the literature. Vaccine. 2012;30(2):121–5. 10.1016/j.vaccine.2011.11.018 . 22100886

[19] Hung IFN, Yuen KY. Immunogenicity, safety and tolerability of intradermal influenza vaccines. Human vaccines & immunotherapeutics. 2018;14(3):565–70. 10.1080/21645515.2017.1328332 28604266 PMC5861844

[20] Combadiere B, Liard C. Transcutaneous and intradermal vaccination. Human vaccines. 2011;7(8):811–27. 10.4161/hv.7.8.16274 . 21817854

[21] Lambert PH, Laurent PE. Intradermal vaccine delivery: will new delivery systems transform vaccine administration? Vaccine. 2008;26(26):3197–208. 10.1016/j.vaccine.2008.03.095 . 18486285

[22] Belyakov IM, Hammond SA, Ahlers JD, Glenn GM, Berzofsky JA. Transcutaneous immunization induces mucosal CTLs and protective immunity by migration of primed skin dendritic cells. Journal of Clinical Investigation. 2004;113(7):998–1007. 10.1172/JCI20261 15057306 PMC379323

[23] Chang SY, Cha HR, Igarashi O, Rennert PD, Kissenpfennig A, Malissen B, et al Cutting Edge: Langerin+ Dendritic Cells in the Mesenteric Lymph Node Set the Stage for Skin and Gut Immune System Cross-Talk. The Journal of Immunology. 2008;180(7):4361–5. 10.4049/jimmunol.180.7.4361 18354155

[24] Zoeteweij JP, Epperson DE, Porter JD, Zhang CX, Frolova OY, Constantinides AP, et al GM1 Binding-Deficient Exotoxin Is a Potent Noninflammatory Broad Spectrum Intradermal Immunoadjuvant. The Journal of Immunology. 2006;177(2):1197–207. 10.4049/jimmunol.177.2.1197 16818778

[25] Guidry JJ, Cardenas L, Cheng E, Clements JD. Role of receptor binding in toxicity, immunogenicity, and adjuvanticity of Escherichia coli heat-labile enterotoxin. Infection and immunity. 1997;65(12):4943–50. Epub 4943. 9393780 10.1128/iai.65.12.4943-4950.1997 PMC175713

[26] White JA, Haghighi C, Brunner J, Estrada M, Lal M, Chen D. Preformulation studies with the Escherichia coli double mutant heat-labile toxin adjuvant for use in an oral vaccine. Journal of immunological methods. 2017;451:83–9. 10.1016/j.jim.2017.09.003 28939395 PMC5703769

[27] Li YF, Poole S, Nishio K, Jang K, Rasulova F, McVeigh A, et al Structure of CFA/I fimbriae from enterotoxigenic Escherichia coli. Proceedings of the National Academy of Sciences of the United States of America. 2009;106(26):10793–8. 10.1073/pnas.0812843106 19515814 PMC2705562

[28] Draize J, Woodard G, Calvery H. Mwethods for the study of irritation and toxicity of substances applied topically to the skin and muous membranes. Journal of Pharmacology and Experimental Therpeutics. 1944;82:377–90.

[29] Li YF, Poole S, Rasulova F, Esser L, Savarino SJ, Xia D. Crystallization and preliminary X-ray diffraction analysis of CfaE, the adhesive subunit of the CFA/I fimbriae from human enterotoxigenic Escherichia coli. Acta crystallographica Section F, Structural biology and crystallization communications. 2006;62(Pt 2):121–4. 10.1107/S1744309105043198 16511280 PMC2150950

[30] Anantha RP, McVeigh AL, Lee LH, Agnew MK, Cassels FJ, Scott DA, et al Evolutionary and functional relationships of colonization factor antigen i and other class 5 adhesive fimbriae of enterotoxigenic Escherichia coli. Infection and immunity. 2004;72(12):7190–201. 10.1128/IAI.72.12.7190-7201.2004 15557644 PMC529125

[31] Chen X, Wu MX. Laser vaccine adjuvant for cutaneous immunization. Expert review of vaccines. 2011;10(10):1397–403. 10.1586/erv.11.112 21988305 PMC3250349

[32] Oreskovic Z, Nechvatalova K, Krejci J, Kummer V, Faldyna M. Aspects of intradermal immunization with different adjuvants: The role of dendritic cells and Th1/Th2 response. PloS one. 2019;14(2):e0211896 10.1371/journal.pone.0211896 30742635 PMC6370205

[33] Carter D, van Hoeven N, Baldwin S, Levin Y, Kochba E, Magill A, et al The adjuvant GLA-AF enhances human intradermal vaccine responses. Sci Adv. 2018;4(9):eaas9930 10.1126/sciadv.aas9930 30221194 PMC6136895

[34] Clements JD, Norton EB. The Mucosal Vaccine Adjuvant LT(R192G/L211A) or dmLT. mSphere. 2018;3(4). 10.1128/mSphere.00215-18 30045966 PMC6060342

[35] Heine SJ, Diaz-McNair J, Andar AU, Drachenberg CB, van de Verg L, Walker R, et al Intradermal delivery of Shigella IpaB and IpaD type III secretion proteins: kinetics of cell recruitment and antigen uptake, mucosal and systemic immunity, and protection across serotypes. Journal of immunology. 2014;192(4):1630–40. 10.4049/jimmunol.1302743 24453241 PMC3998105

[36] Maciel M Jr., Bauer D, Baudier RL, Bitoun J, Clements JD, Poole ST, et al Intradermal or sublingual delivery and heat-labile enterotoxin (LT) proteins shape immunologic outcomes to CFA/I fimbriae-derived subunit antigen vaccine against enterotoxigenic E. coli. Infection and immunity. 2019 10.1128/IAI.00460-19 . 31427449 PMC6803349

[37] Luo Q, Vickers TJ, Fleckenstein JM. Immunogenicity and Protective Efficacy against Enterotoxigenic Escherichia coli Colonization following Intradermal, Sublingual, or Oral Vaccination with EtpA Adhesin. Clinical and vaccine immunology: CVI. 2016;23(7):628–37. 10.1128/CVI.00248-16 27226279 PMC4933781

[38] Liang H, Poncet D, Seydoux E, Rintala ND, Maciel M Jr., Ruiz S, et al The TLR4 agonist adjuvant SLA-SE promotes functional mucosal antibodies against a parenterally delivered ETEC vaccine. NPJ Vaccines. 2019;4:19 10.1038/s41541-019-0116-6 31149350 PMC6538625

[39] Norton EB, Maciel M Jr., Baudier R, Cunningham CK, Janikowski U, Laird RM, et al, editors. Detailed LT antibody analysis on ETEC clinical trial samples 8th Vaccines for Enteric Diseases; 2015; Edinburgh, UK.

[40] Maciel M Jr., Baudier R, Valli E, Mulloy CD, Porter C, Gutierrez RL, et al, editors. Optimizing LT Antibody Analyses to Predict Protection from Oral H10407 ETEC Challenge. 9th Vaccines Against Enteric Diseases; 2017 9-11th 10; Albufeira, Portugal.

[41] Norton EB, Lawson LB, Mahdi Z, Freytag LC, Clements JD. The A subunit of Escherichia coli heat-labile enterotoxin functions as a mucosal adjuvant and promotes IgG2a, IgA, and Th17 responses to vaccine antigens. Infection and immunity. 2012;80(7):2426–35. 10.1128/IAI.00181-12 22526674 PMC3416479

[42] Leach S, Clements JD, Kaim J, Lundgren A. The adjuvant double mutant Escherichia coli heat labile toxin enhances IL-17A production in human T cells specific for bacterial vaccine antigens. PloS one. 2012;7(12):e51718 10.1371/journal.pone.0051718 23284753 PMC3527457

[43] Mitsdoerffer M, Lee Y, Jager A, Kim HJ, Korn T, Kolls JK, et al Proinflammatory T helper type 17 cells are effective B-cell helpers. Proceedings of the National Academy of Sciences of the United States of America. 2010;107(32):14292–7. 10.1073/pnas.1009234107 20660725 PMC2922571

[44] Kuwabara T, Ishikawa F, Kondo M, Kakiuchi T. The Role of IL-17 and Related Cytokines in Inflammatory Autoimmune Diseases. Mediators Inflamm. 2017;2017:3908061 10.1155/2017/3908061 28316374 PMC5337858

