# Supplementary material for: Evaluation of the reactogenicity, adjuvanticity and antigenicity of LT(R192G) and LT(R192G/L211A) by intradermal immunization in mice
Source: PLoS One. 2019 Nov 4;14(11):e0224073. doi: 10.1371/journal.pone.0224073 (PMC6827915; doi:10.1371/journal.pone.0224073)
Supplement: S1 File — (PDF) [file pone.0224073.s001.pdf]

Fig. 2A

Animal #/Study day	10ug dscCfaEB + 2500ng LTR192G							10ug dscCfaEB + 500ng LTR192G							10ug dscCfaEB + 100ng LTR192G						
	#1	#2	#3	#4	#5	#6	#7	#1	#2	#3	#4	#5	#6	#7	#1	#2	#3	#4	#5	#6	#7
1	15.3	13.9	15.6	15.7	16.2	13.7	16.7	11	10.2	10.5	8.46	11.9	12.3	9.47	6.36	9.42	6.49	7.9	7.81	8.24	8.79
2	17	18.6	16.8	17.8	19.2	15.1	15.9	11.2	14.1	16.3	13.1	13.5	14.9	10.7	7.79	4.57	7.96	7.3	8.78	9.02	7.33
3	15.8	13.3	17.8	14.8	16.6	13.2	15.5	12.9	10.2	11.8	11.5	11.7	13.1	8.59	6.12	7.87	6.6	6.84	7.58	7.26	6.68
7	10.1	11	11.2	11.3	10.1	7.59	11.3	9.43	8.56	7.63	7.97	8.4	8.65	8.67	5.91	8.28	6.74	6.37	7.43	7.1	6.71
15	10.4	9.82	7.2	10.6	14.9	8.37	10	8.97	7.07	6.9	6.38	7.91	8.1	6.71	6.58	8.07	5.1	6.41	6.56	6.2	5.89
21	6.61		5.27	0	6.73	0	0	7.03	5.92		0	6.95	0		0			0	0	0	0
28	6.41		4.6	0	5.18	0		4.41	0		0	5.69	0		0			0	0	0	0
35	0		0	0	0	0		0	0		0	0	0		0			0	0	0	0
42	0		0	0	0	0		0	0		0	0	0		0			0	0	0	0

Animal #/Study day	10ug dscCfaEB + 50ng LTR192G							10ug dscCfaEB + 10ng LTR192G							10ug dscCfaEB + 2500ng dmLT						
	#1	#2	#3	#4	#5	#6	#7	#1	#2	#3	#4	#5	#6	#7	#1	#2	#3	#4	#5	#6	#7
1	7.74	6.97	5.91	7.14	8.27	7.73	5.18	5	6.04	5.28	5.74	6.95	4.94	4.63	13.4	15.2	16.7	14.2	13.7	14.1	14.6
2	7.83	6.46	5.58	6.78	8.5	6.42	5.56	5.98	7.04	6.55	5.46	6.03	6.52	5.88	16.8	17.2	20.8	15.8	17	16.2	17.6
3	6.96	5.26	5.38	6.23	6.59	5.42	5.59	5.03	5.79	5.21	5.65	6.51	5.76	5.21	19.8	18.5	17.8	15.6	15.9	19.1	17.1
7	5.28	5.14	5.5	6.33	6.24	5.85	5.18	4	5.1	3.86	5.25	5.26	4.14	4.72	10.2	9.59	9.84	10.3	8.65	9.12	10.4
15	0	4.24	5.58	6.49	6.25	4.75	4.13	0	4.75	0	3.93	0	0	0	9.89	10.8	9.25	9.31	8.22	10.1	9.5
21		0		0	0	0	0	0	0	0		0		0	7.83	6.78	6.61			6.55	8.68
28		0		0	0	0	0	0	0	0		0		0	6.82	6.16	6.63			6.41	6.95
35		0		0	0	0	0	0	0	0		0		0	0	0	0			0	0
42		0		0	0	0	0	0	0	0		0		0	0	0	0			0	0

Fig. 2A

Animal #/Study day	10ug dscCfaEB + 500ng dmLT							10ug dscCfaEB + 100ng dmLT							10ug dscCfaEB + 50ng dmLT						
	#1	#2	#3	#4	#5	#6	#7	#1	#2	#3	#4	#5	#6	#7	#1	#2	#3	#4	#5	#6	#7
1	9.9	8.17	8.96	9.65	8.76	9.4	9.13	5.33	5.36	7.9	6.71	6.77	6.82	6.99	7.16	6.98	3.45	6.09	6.7	6.06	6.11
2	16.8	14.5	16.5	9.65	14.2	13.9	14.3	6.74	6.11	8.64	7.74	8.29	7.42	7.94	6.73	6.89	0	5.69	5.64	5.07	5.95
3	15.4	16	16.2	11.7	16	16.1	16.1	5.97	5.45	6.96	6.68	9.21	7.72	8.55	6.24	6.04	4.41	5.2	5.34	5.3	5.72
7	6.75	6.18	9.98	7.93	7.01	8.47	7.48	5.77	4.92	6.51	5.72	5.2	6.75	7.43	4.6	6.94	0	5.18	5.13	5.49	5.91
15	7.97	6.47	7.55	6.85	6.44	6.25	8.7	6.06	0	0	6.46	4.58	6.33	5.89	0	4.32	0	0	0	4.35	0
21	6.31	6.1	5.97			6.12	7.54		0	4.06		3.32	4.81	5.88			0	0	0	0	0
28	0	5.85	0			5.07	0		0	0		0	0	0			0	0	0	0	0
35	0	0	0			0	0		0	0		0	0	0			0	0	0	0	0
42	0	0	0			0	0		0	0		0	0	0			0	0	0	0	0

Animal #/Study day	10ug dscCfaEB + 10ng dmLT							10ug dscCfaEB							Saline						
	#1	#2	#3	#4	#5	#6	#7	#1	#2	#3	#4	#5	#6	#7	#1	#2	#3	#4	#5	#6	#7
1	3.82	3.68	4.36	0	0	4.91	0	0	0	0	0	0	0	0	0	0	0	0	0	0	0
2	4.38	3.53	4.61	0	0	4.59	0	0	0	0	0	0	0	0	0	0	0	0	0	0	0
3	4.24	0	4.82	0	0	3.68	0	0	0	0	0	0	0	0	0	0	0	0	0	0	0
7	3.53	3.73	3.64	0	0	6.74	0	0	0	0	0	0	0	0	0	0	0	0	0	0	0
15	0	0	0	0	0	0	0	0	0	0	0	0	0	0	0	0	0	0	0	0	0
21	0	0		0	0	0			0	0	0	0	0			0	0	0	0	0	
28	0	0		0	0	0			0	0	0	0	0			0	0	0	0	0	
35	0	0		0	0	0			0	0	0	0	0			0	0	0	0	0	
42	0	0		0	0	0			0	0	0	0	0			0	0	0	0	0	

Fig. 2B

Animal #/Study day	10ug dscCfaEB + 2500ng LTR192G							10ug dscCfaEB + 500ng LTR192G							10ug dscCfaEB + 100ng LTR192G						
	#1	#2	#3	#4	#5	#6	#7	#1	#2	#3	#4	#5	#6	#7	#1	#2	#3	#4	#5	#6	#7
**15**	13.39	14.45	17.7	14.9	18.44	15.32	20.23	11.99	13.2	13.36	11.51	12.79	13.62	11.44	8.15	9.7	10.06	8.875	9.41	10.74	12.27
**16**	15.72	15.79	19.03	13.7	16.19	13.91	18.1	10.39	15.07	11.37	8.765	11.37	14.97	9.67	7.825	7.425	8.1	9.065	8.215	9.77	10.42
**18**	12.3		13.1	9.31	13.15	10.39		8.815	14.84		5.73	8.64	12.06		5.565			6.49	5.31	5.185	6.64
**21**	7.37		6.42	5.88	7.945	9.57		6.815	7.445		6.105	6.965	9.225		5			5.66	5.47	5.235	5.49
**28**	7.82		6.215	6.945	7.95	6.065		7.185	8.605		6.3	6.41	8.235		5.995			4.815	5.755	5.77	5.075
**35**	3.89		0	0	4.905	5.58		3.125	0		3.575	0	0		0			0	0	0	0
**42**	0		0	0	0	0		0	0		0	0	0		0			0	0	0	0

Animal #/Study day	10ug dscCfaEB + 50ng LTR192G							10ug dscCfaEB + 10ng LTR192G							10ug dscCfaEB + 2500ng dmLT						
	#1	#2	#3	#4	#5	#6	#7	#1	#2	#3	#4	#5	#6	#7	#1	#2	#3	#4	#5	#6	#7
**15**	7.72	6.64	7.06	5.91	8.94	8.68	7.195	7.405	7.645	5.81	5.965	6.525	7.065	5.6	11.35	13.39	11.36	12.06	14.3	13.89	12.51
**16**	6.08	5.895	6.8	4.935	8.85	8.465	7.26	6.37	5.245	6.995	5.135	6.17	7.555	5.52	9.69	9.5	9.75	10.51	10.98	16.71	12.1
**18**		5.105		4.265	6.37	6.29	5.15	6.155	4.575	5.475		5.04		4.11	7.36	6.54	7.52			4.94	9.705
**21**		0		0	4.9	4.365	3.935	3.965	4.35	0		4.375		0	6.6	6.7	6.635			6.95	7.44
**28**		0		0	0	4.69	4.55	3.88	0	4.425		4.08		0	6.34	6.39	6.005			8.14	7.22
**35**		0		0	0	0	0	0	0	0		0		0	0	0	0			0	0
**42**		0		0	0	0	0	0	0	0		0		0	0	0	0			0	0

Animal #/Study day	10ug dscCfaEB + 500ng dmLT							10ug dscCfaEB + 100ng dmLT							10ug dscCfaEB + 50ng dmLT						
	#1	#2	#3	#4	#5	#6	#7	#1	#2	#3	#4	#5	#6	#7	#1	#2	#3	#4	#5	#6	#7
**15**	7.475	7.54	8.53	7.655	8.34	8.995	8.135	7.36	7.5	7.5	6.85	7.59	6.64	7.435	4.96	5.515	6.73	6.16	6.12	6.98	5.79
**16**	7.405	7.4	7.68	7.53	6.665	7.745	8.225	7.905	8.725	8.04	6.67	6.075	5.98	7.215	4.605	5.205	5.875	6.79	5.62	5.555	6.89
**18**	4.475	5.405	6.325			5.89	7.265		6.135	4.93		4.675	4.9	5.81			4.27	4.545	4.45	5.095	4.495
**21**	5.245	5.815	5.655			5.283	8		5.65	5.625		4.6	4.895	5			4.66	6.165	5.305	0	4.225
**28**	5.25	4.485	5.165			4.945	4.85		5.835	4.955		4.775	4.52	5.56			4.455	4.87	3.67	0	5.31
**35**	0	0	0			0	0		0	0		0	0	0			0	0	0	0	0
**42**	0	0	0			0	0		0	0		0	0	0			0	0	0	0	0

Animal #/Study day	10ug dscCfaEB + 10ng dmLT							10ug dscCfaEB							Saline						
	#1	#2	#3	#4	#5	#6	#7	#1	#2	#3	#4	#5	#6	#7	#1	#2	#3	#4	#5	#6	#7
**15**	4.77	5.885	4.975	5.9	6.25	6.225	5.925	0	0	0	0	0	0	0	0	0	0	0	0	0	0
**16**	0	5.845	0	6.215	6.89	5.215	5.615	0	0	0	0	0	0	0	0	0	0	0	0	0	0
**18**	0	3.64		4.53	4.445	3.77			0	0	0	0	0			0	0	0	0	0	
**21**	0	3.57		0	4.37	0			0	0	0	0	0			0	0	0	0	0	
**28**	0	0		0	3.94	0			0	0	0	0	0			0	0	0	0	0	
**35**	0	0		0	3.61	0			0	0	0	0	0			0	0	0	0	0	
**42**	0	0		0	0	0			0	0	0	0	0			0	0	0	0	0	

Fig. 2C

Animal #/Study day	10ug dscCfaEB + 2500ng LTR192G							10ug dscCfaEB + 500ng LTR192G							10ug dscCfaEB + 100ng LTR192G						
	#1	#2	#3	#4	#5	#6	#7	#1	#2	#3	#4	#5	#6	#7	#1	#2	#3	#4	#5	#6	#7
**29**	12.5		11.9	10.3	13	12.8		10.4	9.32		10	4.8	9.42		8.68			13.7	8.75	8.33	8.28
**30**	7.44		8.11	6.23	7.88	8.32		6.57	6.2		7.05	5.11	7.34		5.88			5.77	5.28	5.31	4.6
**31**	6.2		5.84	5.61	6.17	5.86		5.31	0		5.42	4.55	4.51		4.23			3.9	4.18	4.46	4.38
**35**	4.53		0	0	5.03	0		4.06	0		4.21	0	3.61		0			0	0	0	4.33
**42**	0		0	0	0	0		0	0		0	0	0		0			0	0	0	0

Animal #/Study day	10ug dscCfaEB + 50ng LTR192G							10ug dscCfaEB + 10ng LTR192G							10ug dscCfaEB + 2500ng dmLT						
	#1	#2	#3	#4	#5	#6	#7	#1	#2	#3	#4	#5	#6	#7	#1	#2	#3	#4	#5	#6	#7
**29**		7.21		6.86	7.31	7.4	7.4	8.36	7.05	7.4		7.95		6.1	9.47	10.2	10.7			9.1	8.66
**30**		4.29		4.56	4.26	4.53	5.16	4.48	5.42	5.36		4.67		4.72	7.26	7.44	9.61			7.93	7.52
**31**		4.69		3.64	3.54	3.55	3.8	3.73	3.89	4.17		0		3.48	6.04	6.83	7.24			4.51	6.76
**35**		0		0	0	0	0	2.74	0	0		0		0	0	4.89	4.81			0	0
**42**		0		0	0	0	0	0	0	0		0		0	0	0	0			0	0

Animal #/Study day	10ug dscCfaEB + 500ng dmLT							10ug dscCfaEB + 100ng dmLT							10ug dscCfaEB + 50ng dmLT						
	#1	#2	#3	#4	#5	#6	#7	#1	#2	#3	#4	#5	#6	#7	#1	#2	#3	#4	#5	#6	#7
**29**	7.97	8.01	3.77			8.62	8.52		7.73	9.2		7.67	8.46	7.1			6.97	7.95	7.5	7.71	7.4
**30**	5.35	5.57	7.12			7.06	6.48		5.1	6.01		5.15	5.61	5.6			4.26	4.61	4.71	5.06	4.86
**31**	4.58	4.55	5.61			5.09	5.29		3.99	4.93		4.31	4.38	4.45			0	4.55	4.09	4.27	0
**35**	3.05	0	0			3.27	2.86		3.1	0		0	0	0			0	0	0	0	0
**42**	0	0	0			0	0		0	0		0	0	0			0	0	0	0	0

Animal #/Study day	10ug dscCfaEB + 10ng dmLT							10ug dscCfaEB							Saline						
	#1	#2	#3	#4	#5	#6	#7	#1	#2	#3	#4	#5	#6	#7	#1	#2	#3	#4	#5	#6	#7
**29**	5.29	4.52		5.25	4.88	7.55			0	0	0	0	0			0	0	0	0	0	
**30**	0	3.67		4.34	0	4.29			0	0	0	0	0			0	0	0	0	0	
**31**	0	0		3.33	0	3.48			0	0	0	0	0			0	0	0	0	0	
**35**	0	0		0	0	0			0	0	0	0	0			0	0	0	0	0	
**42**	0	0		0	0	0			0	0	0	0	0			0	0	0	0	0	

Fig. 2D

Dose and Site/Animal ID	mLT														
	2500 **S1**	2500 **S2**	2500 **S3**	500 **S1**	500 **S2**	500 **S3**	100 **S1**	100 **S2**	100 **S3**	50 **S1**	50 **S2**	50 **S3**	10 **S1**	10 **S2**	10 **S3**
**1**	17.035	15.715	12.45	12.85	11.99	10.435	7.79	8.15	8.68	7.83	7.72		5.975	7.405	8.36
**2**	18.62	15.785		14.08	15.065	9.315	9.42	9.7		6.9735	6.64	7.205	7.04	7.645	7.045
**3**	17.795	19.025	11.86	16.315	13.36		7.955	10.055		5.91	7.06		6.545	6.995	7.395
**4**	17.77	14.895	10.29	13.07	11.51	10.005	7.9	9.065	13.69	7.14	5.91	6.855	5.735	5.965	
**5**	19.16	18.435	13.025	13.525	12.785	5.105	8.78	9.41	8.75	8.495	8.94	7.305	6.945	6.525	7.945
**6**	15.105	15.315	12.765	14.885	14.97	9.415	9.015	10.735	8.325	7.725	8.68	7.395	6.52	7.555	
**7**	16.675	20.225		10.725	11.44		8.79	12.265	8.28	5.585	7.26	7.4	5.88	5.6	6.095

Dose and Site/Animal ID	dmLT														
	d 2500 **S1**	d 2500 **S2**	d 2500 **S3**	d 500**S1**	d 500**S2**	d 500**S3**	d 100**S1**	d 100**S2**	d 100**S3**	d 50 **S1**	d 50 **S2**	d 50 **S3**	d 10 **S1**	d 10 **S2**	d 10 **S3**
**1**	19.83	11.35	9.47	16.82	7.475	7.965	6.735	7.905		7.155	4.96		4.375	4.77	5.29
**2**	18.46	13.385	10.235	15.98	7.54	8.01	6.11	8.725	7.73	6.975	5.515		3.73	5.885	4.52
**3**	20.835	11.355	10.745	16.455	8.53	7.115	8.64	8.04	9.195	4.405	6.73	6.97	4.815	4.975	
**4**	15.815	12.06		11.69	7.655		7.74	6.85		6.085	6.79	7.95	0	6.215	5.245
**5**	16.98	14.3		16.03	8.34		9.21	7.59	7.665	6.695	6.12	7.495	0	6.89	4.88
**6**	19.07	16.705	9.1	16.12	8.995	8.62	7.72	6.64	8.46	6.06	6.98	7.71	6.74	6.225	7.55
**7**	17.6	12.51	8.655	16.085	8.225	8.52	8.55	7.435	7.1	6.11	6.89	7.395	0	5.925	

Fig. 4A

	Group 1					Group 2					Group 3					Group 4				
**Study day/Animal #**	Pool/#1	#2	#3	#4	#5	Pool/#1	#2	#3	#4	#5	Pool/#1	#2	#3	#4	#5	Pool/#1	#2	#3	#4	#5
**0**	1.4					1.4					1.4					1.4				
**14**	3.65					3.07					2.78					2.66				
**28**	4.19					4.54					4.46					4.72				
**42**	4.44	4.34	4.3	4.69	4.71	4.71	5.08	5.01	5.09	4.86	4.19	4.41	4.64	4.39	5.12	4.72	4.93	4.73	5.15	5.26

	Group 5					Group 6					Group 7					Group 8				
**Study day/Animal #**	Pool/#1	#2	#3	#4	#5	Pool/#1	#2	#3	#4	#5	Pool/#1	#2	#3	#4	#5	Pool/#1	#2	#3	#4	#5
**0**	1.4					1.4					1.4					1.4				
**14**	3.16					3.27					3.31					3.14				
**28**	4.54					4.8					4.84					4.85				
**42**	4.7	4.46	4.99	5.27	5.25	4.88	4.77	4.84	4.93	5.02	4.92	5.1	4.98	4.92	5.54	4.72	4.77	4.57	4.98	5.32

	Group 9					Group 10					Group 11					Group 12				
**Study day/Animal #**	Pool/#1	#2	#3	#4	#5	Pool/#1	#2	#3	#4	#5	Pool/#1	#2	#3	#4	#5	Pool/#1	#2	#3	#4	#5
**0**	1.4					1.4					1.4					1.4				
**14**	3.39					3.22					2.03					1.4				
**28**	5.07					4.87					2.94					1.4				
**42**	5.53	5.33	5.36	5.33	5.35	5.5	4.69	5.51	5.35	4.9	2.2	3.18	4.04	2.59	3.01	1.4	1.4	1.4	1.4	1.4

Groups ID	
**Group 1**	10 mcg CfaE + 0.01 mcg mLT
**Group 2**	10 mcg CfaE + 0.05 mcg mLT
**Group 3**	10 mcg CfaE + 0.1 mcg mLT
**Group 4**	10 mcg CfaE + 0.5 mcg mLT
**Group 5**	10 mcg CfaE + 2.5 mcg mLT
**Group 6**	10 mcg CfaE + 0.01 mcg dmLT
**Group 7**	10 mcg CfaE + 0.05 mcg dmLT
**Group 8**	10 mcg CfaE + 0.1 mcg dmLT
**Group 9**	10 mcg CfaE + 0.5 mcg dmLT
**Group 1**	10 mcg CfaE + 2.5 mcg dmLT
**Group 1**	10 mcg CfaE
**Group 1**	Saline

Fig. 4B

Animal #	Group 1	Group 2	Group 3	Group 4	Group 5	Group 6	Group 7	Group 8	Group 9	Group 10	Group 11	Group 12
1	4.44	4.71	4.19	4.72	4.7	4.88	4.92	4.72	5.53	5.5	2.2	1.4
2	4.34	5.08	4.41	4.93	4.46	4.77	5.1	4.77	5.33	4.69	3.18	1.4
3	4.3	5.01	4.64	4.73	4.99	4.84	4.98	4.57	5.36	5.51	4.04	1.4
4	4.69	5.09	4.39	5.15	5.27	4.93	4.92	4.98	5.33	5.35	2.59	1.4
5	4.71	4.86	5.12	5.26	5.25	5.02	5.54	5.32	5.35	4.9	3.01	1.4

Groups ID	
**Group 1:**	10 mcg CfaE + 0.01 mcg mLT
**Group 2:**	10 mcg CfaE + 0.05 mcg mLT
**Group 3:**	10 mcg CfaE + 0.1 mcg mLT
**Group 4:**	10 mcg CfaE + 0.5 mcg mLT
**Group 5:**	10 mcg CfaE + 2.5 mcg mLT
**Group 6:**	10 mcg CfaE + 0.01 mcg dmLT
**Group 7:**	10 mcg CfaE + 0.05 mcg dmLT
**Group 8:**	10 mcg CfaE + 0.1 mcg dmLT
**Group 9:**	10 mcg CfaE + 0.5 mcg dmLT
**Group 10:**	10 mcg CfaE + 2.5 mcg dmLT
**Group 11:**	10 mcg CfaE
**Group 12:**	Saline

Fig. 4C

	IgG1											
**Duplicates**	**2500 mLT G1**	**500 mLT G1**	**100 mLT G1**	**50 mLT G1**	**10 mLT G1**	**2500 dmLT G1**	**500 dmLT g1**	**100 dmLT G1**	**50 dmLT G1**	**10 dmLT g1**	**CfaEB G1**	**Saline G1**
**Assay 1**	5.535	5.588	5.539	5.667	5.678	5.63	5.694	5.613	5.72	5.684	4.168	1.916
**Assay 2**	5.561	5.553	5.475	5.583	5.565	5.522	5.523	5.594	5.644	5.6	4.154	1.683

	IgG2a											
**Duplicates**	**2500 mLT 2a**	**500 mLT 2a**	**100 mLT 2a**	**50 mLT 2a**	**10 mLT 2a**	**2500 dmLT 2a**	**500 dmLT 2a**	**100 dmLT 2a**	**50 dmLT 2a**	**10 dmLT 2a**	**CfaEB 2a**	**Saline 2a**
**Assay 1**	3.818	3.393	3.03	2.839	3.224	3.9	3.587	3.361	3.288	3.015	1.4	1.4
**Assay 2**	3.836	3.488	3.13	2.957	3.278	3.804	3.427	3.293	3.153	2.996	1.4	1.4

Fig. 4D

Duplicates	Group 1	Group 2	Group 3	Group 4	Group 5	Group 6	Group 7	Group 8	Group 9	Group 10	Group 11	Group 12
**Assay 1**	3.31	3.11	2.93	2.88	3.06	3.42	3.38	3.34	3.33	3.37	1.4	1.4
**Assay 2**	3.33	3.1	3.01	2.96	3.09	3.47	3.44	3.29	3.38	3.11	1.72	1.4

Groups ID	
**Group 1:**	10 mcg CfaE + 0.01 mcg mLT
**Group 2:**	10 mcg CfaE + 0.05 mcg mLT
**Group 3:**	10 mcg CfaE + 0.1 mcg mLT
**Group 4:**	10 mcg CfaE + 0.5 mcg mLT
**Group 5:**	10 mcg CfaE + 2.5 mcg mLT
**Group 6:**	10 mcg CfaE + 0.01 mcg dmLT
**Group 7:**	10 mcg CfaE + 0.05 mcg dmLT
**Group 8:**	10 mcg CfaE + 0.1 mcg dmLT
**Group 9:**	10 mcg CfaE + 0.5 mcg dmLT
**Group 10:**	10 mcg CfaE + 2.5 mcg dmLT
**Group 11:**	10 mcg CfaE
**Group 12:**	Saline

Fig. 4E

Animal #	Group 1	Group 2	Group 3	Group 4	Group 5	Group 6	Group 7	Group 8	Group 9	Group 10	Group 11	Group 12
**1**	1.97	2.62	2.68	2.78	2.64	2.56	3.06	2.63	3.69	2.83	0.88	0.88
**2**	2.49	2.4	2.12	2.21	2.7	3.34	2.66	3.08	3.28	1.95	0.88	0.88
**3**	2.52	2.35	2.25	1.62	2.9	2.87	3.06	2.92	2.93	3.43	0.88	0.88
**4**	2.09	2.48	2.58	2.32	2.36	3.3	3.26	3.32	2.04	3.54	0.88	0.88
**5**	2.81	2.59	2.77	2.83	2.41	2.4	3.21	2.92	2.56	1.9	0.88	0.88

Groups ID	
**Group 1:**	10 mcg CfaE + 0.01 mcg mLT
**Group 2:**	10 mcg CfaE + 0.05 mcg mLT
**Group 3:**	10 mcg CfaE + 0.1 mcg mLT
**Group 4:**	10 mcg CfaE + 0.5 mcg mLT
**Group 5:**	10 mcg CfaE + 2.5 mcg mLT
**Group 6:**	10 mcg CfaE + 0.01 mcg dmLT
**Group 7:**	10 mcg CfaE + 0.05 mcg dmLT
**Group 8:**	10 mcg CfaE + 0.1 mcg dmLT
**Group 9:**	10 mcg CfaE + 0.5 mcg dmLT
**Group 10:**	10 mcg CfaE + 2.5 mcg dmLT
**Group 11:**	10 mcg CfaE
**Group 12:**	Saline

Fig. 5A

Animal #	Group 1	Group 2	Group 3	Group 4	Group 5	Group 6	Group 7	Group 8	Group 9	Group 10	Group 11	Group 12
**1**	4.986	5.056	4.87	4.879	5.492	5.106	5.581	5.306	5.884	4.762	1.98	1.4
**2**	5.046	5.605	5.216	5.076	4.74	5.187	5.095	5.925	4.906	4.661	1.4	1.4
**3**	5.091	5.047	5.215	5.081	5.128	5.137	5.362	6.005	5.115	5.084	4.394	1.4
**4**	5.072	5.295	5.237	5.308	5.199	5.405	5.152	5.715	5.635	4.721	2.367	1.4
**5**	5.48	5.02	4.974	5.529	5.158	5.381	5.136	5.875	5.052	5.835	1.961	1.4

Groups ID	
**Group 1:**	10 mcg CfaE + 0.01 mcg mLT
**Group 2:**	10 mcg CfaE + 0.05 mcg mLT
**Group 3:**	10 mcg CfaE + 0.1 mcg mLT
**Group 4:**	10 mcg CfaE + 0.5 mcg mLT
**Group 5:**	10 mcg CfaE + 2.5 mcg mLT
**Group 6:**	10 mcg CfaE + 0.01 mcg dmLT
**Group 7:**	10 mcg CfaE + 0.05 mcg dmLT
**Group 8:**	10 mcg CfaE + 0.1 mcg dmLT
**Group 9:**	10 mcg CfaE + 0.5 mcg dmLT
**Group 10:**	10 mcg CfaE + 2.5 mcg dmLT
**Group 11:**	10 mcg CfaE
**Group 12:**	Saline

Fig. 5B

Animal #	Group 1	Group 2	Group 3	Group 4	Group 5	Group 6	Group 7	Group 8	Group 9	Group 10	Group 11	Group 12
**1**	8192	4096	2048	3072	4096	2048	4096	4096	4096	3072	8	8
**2**	8192	8192	2048	3072	2048	3072	3072	4096	4096	1024	32	
**3**	6144	8192	4096	4096	4096	3072	4096	6144	6144	1024	24	
**4**	8192	8192	2048	8192	4096	6144	4096	4096	6144	1024	16	
**5**	8192	6144	3072	8192	4096	4096	3072	2048	6144	2048	32	

**Groups ID**	
**Group 1:**	10 mcg CfaE + 0.01 mcg mLT
**Group 2:**	10 mcg CfaE + 0.05 mcg mLT
**Group 3:**	10 mcg CfaE + 0.1 mcg mLT
**Group 4:**	10 mcg CfaE + 0.5 mcg mLT
**Group 5:**	10 mcg CfaE + 2.5 mcg mLT
**Group 6:**	10 mcg CfaE + 0.01 mcg dmLT
**Group 7:**	10 mcg CfaE + 0.05 mcg dmLT
**Group 8:**	10 mcg CfaE + 0.1 mcg dmLT
**Group 9:**	10 mcg CfaE + 0.5 mcg dmLT
**Group 10:**	10 mcg CfaE + 2.5 mcg dmLT
**Group 11:**	10 mcg CfaE
**Group 12:**	Saline

Fig. 6A

	Group 1					Group 2					Group 3					Group 4				
**Study day/Animal #**	Pool/#1	#2	#3	#4	#5	Pool/#1	#2	#3	#4	#5	Pool/#1	#2	#3	#4	#5	Pool/#1	#2	#3	#4	#5
**0**	1.4					1.4					1.4					1.4				
**14**	4.27					4.1					3.54					3.55				
**28**	4.89					5.41					4.85					4.88				
**42**	4.9	5.51	4.94	4.88	4.87	5.42	4.99	4.78	5.63	4.41	5.48	5.53	4.88	4.44	4.97	4.93	4.36	5	4.7	4.67

	Group 5					Group 6					Group 7					Group 8				
**Study day/Animal #**	Pool/#1	#2	#3	#4	#5	Pool/#1	#2	#3	#4	#5	Pool/#1	#2	#3	#4	#5	Pool/#1	#2	#3	#4	#5
**0**	1.4					1.4					1.4					1.4				
**14**	4.09					4.65					4.65					3.73				
**28**	4.41					5.68					5.43					5.3				
**42**	4.55	5.44	4.95	4.33	4.92	5.53	5.6	5.8	5.82	6.06	5.33	4.86	5.64	5.5	5.35	5.74	4.7	5.74	5.61	5.63

	Group 9					Group 10					Group 11					Group 12				
**Study day/Animal #**	Pool/#1	#2	#3	#4	#5	Pool/#1	#2	#3	#4	#5	Pool/#1	#2	#3	#4	#5	Pool/#1	#2	#3	#4	#5
**0**	1.4					1.4					1.4					1.4				
**14**	3.79					3.52					1.4					1.4				
**28**	5.3					4.73					1.4					1.4				
**42**	5.73	5.27	5.34	5.6	4.79	4.63	4.54	4.91	4.93	4.74	1.4	1.4	1.4	1.4	1.4	1.4	1.4	1.4	1.4	1.4

Groups ID	
**Group 1:**	10 mcg CfaE + 0.01 mcg mLT
**Group 2:**	10 mcg CfaE + 0.05 mcg mLT
**Group 3:**	10 mcg CfaE + 0.1 mcg mLT
**Group 4:**	10 mcg CfaE + 0.5 mcg mLT
**Group 5:**	10 mcg CfaE + 2.5 mcg mLT
**Group 6:**	10 mcg CfaE + 0.01 mcg dmLT
**Group 7:**	10 mcg CfaE + 0.05 mcg dmLT
**Group 8:**	10 mcg CfaE + 0.1 mcg dmLT
**Group 9:**	10 mcg CfaE + 0.5 mcg dmLT
**Group 1(**	10 mcg CfaE + 2.5 mcg dmLT
**Group 1:**	10 mcg CfaE
**Group 1:**	Saline

Fig. 6B

Animal #	Group 1	Group 2	Group 3	Group 4	Group 5	Group 6	Group 7	Group 8	Group 9	Group 10	Group 11	Group 12
**1**	4.9	5.42	5.48	4.93	4.55	5.53	5.33	5.74	5.73	4.63	1.4	1.4
**2**	5.51	4.99	5.53	4.36	5.44	5.6	4.86	4.7	5.27	4.54	1.4	1.4
**3**	4.94	4.78	4.88	5	4.95	5.8	5.64	5.74	5.34	4.91	1.4	1.4
**4**	4.88	5.63	4.44	4.7	4.33	5.82	5.5	5.61	5.6	4.93	1.4	1.4
**5**	4.87	4.41	4.97	4.67	4.92	6.06	5.35	5.63	4.79	4.74	1.4	1.4

Groups ID	
**Group 1:**	10 mcg CfaE + 0.01 mcg mLT
**Group 2:**	10 mcg CfaE + 0.05 mcg mLT
**Group 3:**	10 mcg CfaE + 0.1 mcg mLT
**Group 4:**	10 mcg CfaE + 0.5 mcg mLT
**Group 5:**	10 mcg CfaE + 2.5 mcg mLT
**Group 6:**	10 mcg CfaE + 0.01 mcg dmLT
**Group 7:**	10 mcg CfaE + 0.05 mcg dmLT
**Group 8:**	10 mcg CfaE + 0.1 mcg dmLT
**Group 9:**	10 mcg CfaE + 0.5 mcg dmLT
**Group 10:**	10 mcg CfaE + 2.5 mcg dmLT
**Group 11:**	10 mcg CfaE
**Group 12:**	Saline

Fig. 6C

Duplicates	IgG1											
	2500 mLT	500 mLT	100 mLT	50 mLT	10 mLT	2500 dmLT	500 dmLT	100 dmLT	50 dmLT	10 dmLT	CfaEB	Saline
Assay 1	5.646	5.576	5.563	5.497	5.42	5.82	5.574	5.678	5.6	5.468	2.95	2.567
Assay 2	5.536	5.497	5.52	5.448	5.38	5.726	5.492	5.549	5.526	5.395	2.964	2.77

IgG2a											
2500 mLT	500 mLT	100 mLT	50 mLT	10 mLT	2500 dmLT	500 dmLT	100 dmLT	50 dmLT	10 dmLT	CfaEB	Saline
4.722	4.643	3.78	3.714	3.68	4.842	4.479	4.649	4.706	3.61	1.4	1.4
4.771	4.609	3.788	3.709	3.67	4.926	4.459	4.646	4.672	3.676	1.4	1.4

Fig. 7A

IFN-gamma

	CfaE-specific											
**Duplicates**	2500 mLT CfaE	2500 dmLT CfaE	500 mLT CfaE	500 dmLT CfaE	100 mLT CfaE	100 dmLT CfaE	50 mLT CfaE	50 dmLT CfaE	10 mLT CfaE	10 dmLT CfaE	dscCfaEB CfaE	Saline CfaE
**Assay 1**	89.66	44.01	164.02	11.31	187.79	1.94	128.95	1.87	14.51	8.65	0.24	0.15
**Assay 2**	108.94	45.93	193.81	11.54	196.34	2.11	171.16	2.1	22.82	6.35	0.41	0.05

	LTB-specific											
**Duplicates**	2500 mLT LTB	2500 dmLT LTB	500 mL LTB	500 dmLT LTB	100 mLT LTB	100 dmLT LTB	50 mLT LTB	50 dmLT LTB	10 mLT LTB	10 dmLT LTB	dscCfaEB LTB	Saline LTB
**Assay 1**	0.45	23.48	0.27	1.79	0.57	1.23	0.67	2.64	0	1.81	0	0
**Assay 2**	0.61	32.83	0.62	1.68	0.91	1.55	0.3	1.62	0.21	2.44	0.05	0.18

Fig. 7B

IL-4

	CfaE-specific											
**Duplicates**	2500 mLT CfaE	2500 dmLT CfaE	500 mLT CfaE	500 dmLT CfaE	100 mLT CfaE	100 dmLT CfaE	50 mLT CfaE	50 dmLT CfaE	10 mLT CfaE	10 dmLT CfaE	dscCfaEB CfaE	Saline CfaE
**Assay 1**	145.03	279.72	305.06	80.04	121.89	131.63	223.79	145.03	133.78	109.15	2.11	0.1
**Assay 2**	147.95	305.29	355.65	84.11	124.57	140.83	219.36	160.75	137.38	118.14	2.39	0.08

	LTB-specific											
**Duplicates**	2500 mLT LTB	2500 dmLT LTB	500 mL LTB	500 dmLT LTB	100 mLT LTB	100 dmLT LTB	50 mLT LTB	50 dmLT LTB	10 mLT LTB	10 dmLT LTB	dscCfaEB LTB	Saline LTB
**Assay 1**	10.93	31.51	14.03	18.7	9.8	8.91	5.26	10.88	8.1	5.87	0.32	0.1
**Assay 2**	11.32	35.97	15.38	21.24	10.27	8.96	6.29	13.83	10.19	7.01	0.36	0.11

Fig. 7C

IL-5

	CfaE-specific											
**Duplicates**	2500 mLT CfaE	2500 dmLT CfaE	500 mLT CfaE	500 dmLT CfaE	100 mLT CfaE	100 dmLT CfaE	50 mLT CfaE	50 dmLT CfaE	10 mLT CfaE	10 dmLT CfaE	dscCfaEB CfaE	Saline CfaE
**Assay 1**	915.42	1132.31	1978.78	709.46	1265.85	1056.77	2574.91	868.94	2644.47	1379.95	37.82	0
**Assay 2**	985.49	1386.54	2274.14	711.54	1300.31	1057.18	2653.17	1063.84	2724.97	1621.22	45.82	0

LTB-specific											
2500 mLT LTB	2500 dmLT LTB	500 mL LTB	500 dmLT LTB	100 mLT LTB	100 dmLT LTB	50 mLT LTB	50 dmLT LTB	10 mLT LTB	10 dmLT LTB	dscCfaEB LTB	Saline LTB
21.11	92.86	185.61	74.21	240.12	65.82	82.72	66.62	155.33	32.42	0	0
24.04	120.23	235.96	88.38	250.77	63.8	91.84	82.86	167.19	36.85	0	0

Fig. 7D

IL-10

	CfaE-specific											
**Duplicates**	2500 mLT CfaE	2500 dmLT CfaE	500 mLT CfaE	500 dmLT CfaE	100 mLT CfaE	100 dmLT CfaE	50 mLT CfaE	50 dmLT CfaE	10 mLT CfaE	10 dmLT CfaE	dscCfaEB CfaE	Saline CfaE
**Assay 1**	202.71	312.14	415.47	156.89	249.65	177.05	296.99	114.27	305.9	159.63	0.22	0
**Assay 2**	211.05	392.6	444.19	166.11	255.4	198	294.06	134.83	326.91	190.73	0	0

	LTB-specific											
**Duplicates**	2500 mLT LTB	2500 dmLT LTB	500 mL LTB	500 dmLT LTB	100 mLT LTB	100 dmLT LTB	50 mLT LTB	50 dmLT LTB	10 mLT LTB	10 dmLT LTB	dscCfaEB LTB	Saline LTB
**Assay 1**	7.27	26.66	15.19	12.03	9.45	8.44	6.61	9.54	6.86	2.18	0	0
**Assay 2**	8.1	38.68	16.26	9.79	10.48	7.6	8.1	11.51	9.45	2.63	0	0

Fig. 7E

IL-17

	CfaE-specific											
**Duplicates**	2500 mLT CfaE	2500 dmLT CfaE	500 mLT CfaE	500 dmLT CfaE	100 mLT CfaE	100 dmLT CfaE	50 mLT CfaE	50 dmLT CfaE	10 mLT CfaE	10 dmLT CfaE	dscCfaEB CfaE	Saline CfaE
**Assay 1**	28.16	23.64	29.38	0	13.51	0	9.69	0	0	0	0	0
**Assay 2**	27.07	32.08	34.34	0	14.2	0	10.28	0	0	0	0	0

	LTB-specific											
**Duplicates**	2500 mLT LTB	2500 dmLT LTB	500 mL LTB	500 dmLT LTB	100 mLT LTB	100 dmLT LTB	50 mLT LTB	50 dmLT LTB	10 mLT LTB	10 dmLT LTB	dscCfaEB LTB	Saline LTB
**Assay 1**	0	0	0	0	17.71	82.92	45.61	0	151.13	143.93	0	0
**Assay 2**	0	0	0	0	16.16	84.05	54.4	0	217.8	189.84	0	0

Suppl Fig. 2A-F

Erythema site 1: Suppl. 2A

	10ug dscCfaEB + 10ng dmLT/mLT							10ug dscCfaEB + 50ng dmLT/mLT							10ug dscCfaEB + 100ngdmLT/mLT							10ug dscCfaEB + 500ng dmLT/mLT							10ug dscCfaEB + 2500ng dmLT/mLT							10ug dscCfaEB							Saline						
**Adj./Animal #**	#1	#2	#3	#4	#5	#6	#7	#1	#2	#3	#4	#5	#6	#7	#1	#2	#3	#4	#5	#6	#7	#1	#2	#3	#4	#5	#6	#7	#1	#2	#3	#4	#5	#6	#7	#1	#2	#3	#4	#5	#6	#7	#1	#2	#3	#4	#5	#6	#7
**dmLT**	0	0	0	0	0	0	0	1	1	0	1	1	1	1	1	1	1	1	1	2	1	1	1	1	2	1	1	1	2	3	3	1	2	2	1	0	0	0	0	0	0	0	0	0	0	0	0	0	0
**mLT**	0	1	0	1	0	0	0	1	1	1	1	2	1	1	1	1	1	1	1	1	1	2	1	1	1	2	2	2	2	2	2	1	2	2	2	0	0	0	0	0	0	0	0	0	0	0	0	0	0

Erythema site 2: Suppl. 2B

**Adj./Animal #**	10ug dscCfaEB + 10ng dmLT/mLT							10ug dscCfaEB + 50ng dmLT/mLT							10ug dscCfaEB + 100ngdmLT/mLT							10ug dscCfaEB + 500ng dmLT/mLT							10ug dscCfaEB + 2500ng dmLT/mLT							10ug dscCfaEB							Saline						
**dmLT**	#1	#2	#3	#4	#5	#6	#7	#1	#2	#3	#4	#5	#6	#7	#1	#2	#3	#4	#5	#6	#7	#1	#2	#3	#4	#5	#6	#7	#1	#2	#3	#4	#5	#6	#7	#1	#2	#3	#4	#5	#6	#7	#1	#2	#3	#4	#5	#6	#7
**mLT**	0	0	0	0	0	0	0	0	0	0	1	1	0	0	1	1	0	1	1	1	1	2	2	2	2	2	1	2	2	2	2	2	2	1	2	0	0	0	0	0	0	0	0	0	0	0	0	0	0
	0	0	0	1	1	1	0	0	1	1	0	0	1	1	1	2	1	1	1	1	1	1	1	1	2	1	1	2	2	2	2	1	2	2	1	0	0	0	0	0	0	0	0	0	0	0	0	0	0

Erythema site 3: Suppl. 2C

	10ug dscCfaEB + 10ng dmLT/mLT							10ug dscCfaEB + 50ng dmLT/mLT							10ug dscCfaEB + 100ngdmLT/mLT							10ug dscCfaEB + 500ng dmLT/mLT							10ug dscCfaEB + 2500ng dmLT/mLT							10ug dscCfaEB							Saline						
**Adj./Animal #**	#1	#2	#3	#4	#5	#6	#7	#1	#2	#3	#4	#5	#6	#7	#1	#2	#3	#4	#5	#6	#7	#1	#2	#3	#4	#5	#6	#7	#1	#2	#3	#4	#5	#6	#7	#1	#2	#3	#4	#5	#6	#7	#1	#2	#3	#4	#5	#6	#7
**dmLT**	1	2	1	1	2	1	1	1	2	2	1	2	1	2	2	2	2	2	2	2	2	2	2	2	2	2	2	2	2	2	2	2	2	1	2	0	0	0	0	0	0	0	0	0	0	0	0	0	0
**mLT**	2	1	2	1	2	2	1	1	2	2	1	1	2	1	2	2	2	2	2	2	2	2	1	2	2	2	1	2	2	2	2	2	2	2	2	0	0	0	0	0	0	0	0	0	0	0	0	0	0

Edema site 1: Suppl. 2D

	10ug dscCfaEB + 10ng dmLT/mLT							10ug dscCfaEB + 50ng dmLT/mLT							10ug dscCfaEB + 100ngdmLT/mLT							10ug dscCfaEB + 500ng dmLT/mLT							10ug dscCfaEB + 2500ng dmLT/mLT							10ug dscCfaEB							Saline						
**Adj./Animal #**	#1	#2	#3	#4	#5	#6	#7	#1	#2	#3	#4	#5	#6	#7	#1	#2	#3	#4	#5	#6	#7	#1	#2	#3	#4	#5	#6	#7	#1	#2	#3	#4	#5	#6	#7	#1	#2	#3	#4	#5	#6	#7	#1	#2	#3	#4	#5	#6	#7
**dmLT**	1	1	1	0	0	2	0	2	2	1	2	2	2	2	2	2	2	2	2	2	2	1	1	1	2	2	2	1	2	3	3	1	3	1	1	0	0	0	0	0	0	0	0	0	0	0	0	0	0
**mLT**	1	1	1	2	1	2	1	2	1	2	2	2	2	1	2	3	2	2	2	2	2	2	2	2	2	2	2	3	2	2	2	1	2	2	2	0	0	0	0	0	0	0	0	0	0	0	0	0	0

Edema site 2: Suppl. 2E

	10ug dscCfaEB + 10ng dmLT/mLT							10ug dscCfaEB + 50ng dmLT/mLT							10ug dscCfaEB + 100ngdmLT/mLT							10ug dscCfaEB + 500ng dmLT/mLT							10ug dscCfaEB + 2500ng dmLT/mLT							10ug dscCfaEB							Saline						
**Adj./Animal #**	#1	#2	#3	#4	#5	#6	#7	#1	#2	#3	#4	#5	#6	#7	#1	#2	#3	#4	#5	#6	#7	#1	#2	#3	#4	#5	#6	#7	#1	#2	#3	#4	#5	#6	#7	#1	#2	#3	#4	#5	#6	#7	#1	#2	#3	#4	#5	#6	#7
**dmLT**	1	2	1	1	2	1	1	1	2	2	1	2	1	2	2	2	2	2	2	2	2	2	2	2	2	2	2	2	2	2	2	2	2	1	2	0	0	0	0	0	0	0	0	0	0	0	0	0	0
**mLT**	2	1	2	1	2	2	1	1	2	2	1	1	2	1	2	2	2	2	2	2	2	2	1	2	2	2	1	2	2	2	2	2	2	2	2	0	0	0	0	0	0	0	0	0	0	0	0	0	0

Edema site 3: Suppl. 2F

	10ug dscCfaEB + 10ng dmLT/mLT							10ug dscCfaEB + 50ng dmLT/mLT							10ug dscCfaEB + 100ngdmLT/mLT							10ug dscCfaEB + 500ng dmLT/mLT							10ug dscCfaEB + 2500ng dmLT/mLT							10ug dscCfaEB							Saline						
**Adj./Animal #**	#1	#2	#3	#4	#5	#6	#7	#1	#2	#3	#4	#5	#6	#7	#1	#2	#3	#4	#5	#6	#7	#1	#2	#3	#4	#5	#6	#7	#1	#2	#3	#4	#5	#6	#7	#1	#2	#3	#4	#5	#6	#7	#1	#2	#3	#4	#5	#6	#7
**dmLT**	1	1		1	1	1	0	0	0	1	1	1	1	1		1	2		1	1	2	2	2	2			2	2	2	2	2	0	0	2	2	0	0	0	0	0	0			0	0	0	0	0	0
**mLT**	1	1	1		1	0	1	0	1		1	1	1	1	1			1	1	1	1	2	1		2	1	2	0	1		1	1	1	1		0	0	0	0	0	0	0	0	0	0	0	0	0	0

Suppl Fig. 3A-C

Skin pathology scores SD16: Suppl. 3A

Adjuvant/Dose & site	2500 S1	2500 S2	2500 S3	500 S1	500 S2	500 S3	100 S1	100 S2	100 S3	50 S1	50 S2	50 S3	10 S1	10 S2	10 S3
mLT	2	4	0	3	4	0	2	3	0	2	4	0	1	4	0
dmLT	2	4	0	3	4	0	3	4	0	3	4	0	1	3	0

Adjuvant/Dose & site	2500 S1	2500 S2	2500 S3	500S1	500S2	500S3	100S1	100S2	100S3	50 S1	50 S2	50 S3	10 S1	10 S2	10 S3
mLT	1	3	0	3	4	0	3	4	0	1	3	0	1	2	0
dmLT	3	4	0	3	4	0	1	4	0	2	3	0	0	2	0

Skin pathology scores SD42: Suppl. 3B

Adjuvant/Dose & site	2500 S1	2500 S2	2500 S3	500 S1	500 S2	500 S3	100 S1	100 S2	100 S3	50 S1	50 S2	50 S3	10 S1	10 S2	10 S3
mLT	0	0	0	0	0	4	0	0	2	0	0	2	0	0	0
dmLT	0	1	2	0	0	3	0	0	2	1	0	0	0	0	2

Adjuvant/Dose & site	2500 S1	2500 S2	2500 S3	500S1	500S2	500S3	100S1	100S2	100S3	50 S1	50 S2	50 S3	10 S1	10 S2	10 S3
mLT	0	0	2	0	0	2	1	0	2	0	0	0	0	0	2
dmLT	0	0	1	0	0	2	0	0	1	0	0	2	0	0	3

Edema SD16: Suppl. 3C

Adjuvant/Dose & site	2500 S1	2500 S2	2500 S3	500 S1	500 S2	500 S3	100 S1	100 S2	100 S3	50 S1	50 S2	50 S3	10 S1	10 S2	10 S3
mLT	1	4	0	1	3	0	1	1	0	1	4	0	1	3	0
dmLT	1	4	0	1	5	0	2	4	0	1	4	0	1	3	0

Adjuvant/Dose & site	2500 S1	2500 S2	2500 S3	500S1	500S2	500S3	100S1	100S2	100S3	50 S1	50 S2	50 S3	10 S1	10 S2	10 S3
mLT	1	2	0	2	4	0	2	4	0	1	3	0	1	1	0
dmLT	2	4	0	2	4	0	1	4	0	1	3	0	0	1	0
