# Supplementary figures and images for: Evaluation of the reactogenicity, adjuvanticity and antigenicity of LT(R192G) and LT(R192G/L211A) by intradermal immunization in mice
Source: PLoS One. 2019 Nov 4;14(11):e0224073. doi: 10.1371/journal.pone.0224073 (PMC6827915; doi:10.1371/journal.pone.0224073)

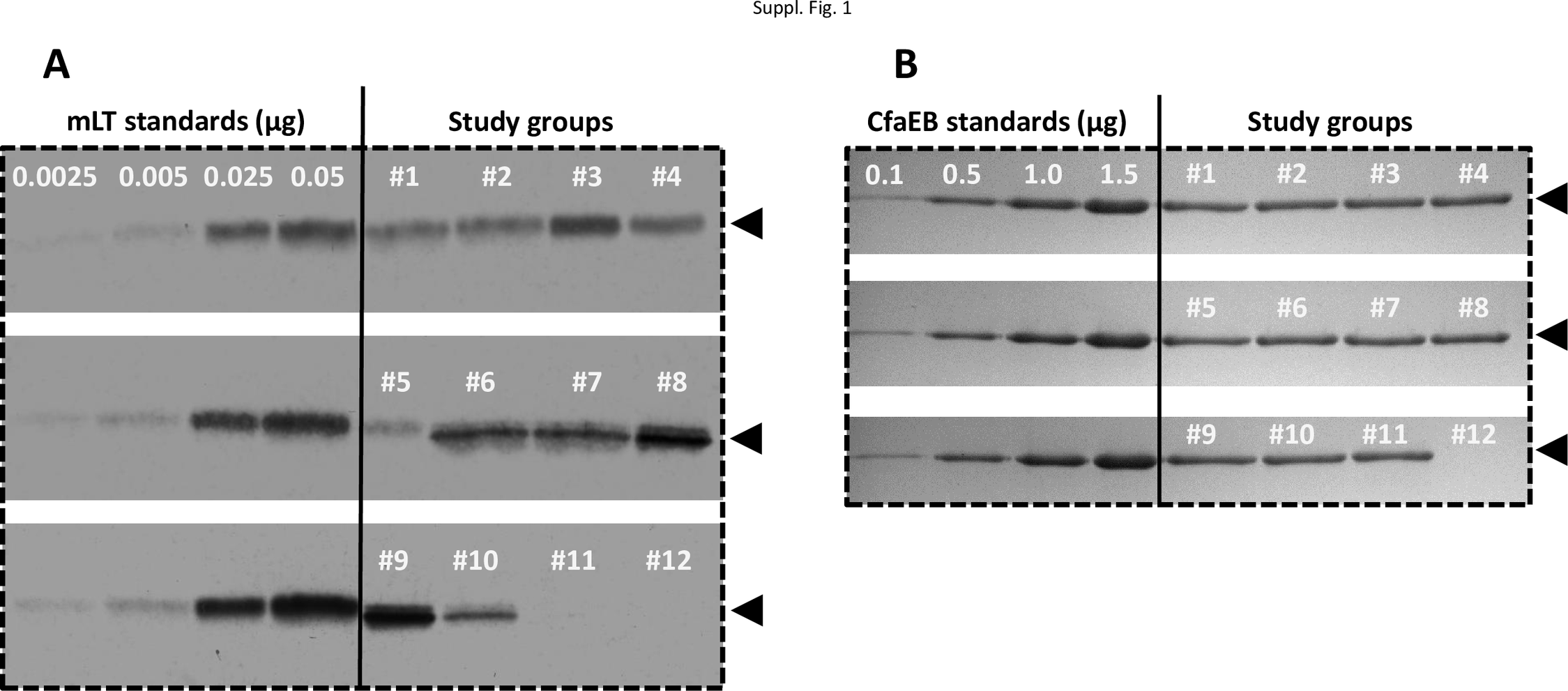

Supplement: S1 Fig — (A) Western blot analysis of mLT standards (0.0025, 0.005, 0.025, and 0.05 μg) and dose formulations for Study Groups 1–12 using rabbit polyclonal sera generated against the B subunit of LT. The expected amounts of mLT/dmLT for the analysis based on the original concentration of each group’s formulation are as follows: #1–0.025 μg (100-fold dilution of the original 2.5 μg of mLT); #2–0.025 μg (20-fold dilution of the original 0.5 μg of mLT); #3–0.05 μg (2-fold dilution of the original 0.1 μg of mLT); #4–0.025 μg (2-fold dilution of the original 0.05 μg of mLT); #5–0.005 μg (2-fold dilution of the original 0.01 μg of mLT); #6–0.025 μg (100-fold dilution of the original 2.5 μg of dmLT); #7–0.025 μg (20-fold dilution of the original 0.5 μg of dmLT); #8–0.05 μg (2-fold dilution of the original 0.1 μg of dmLT); #9–0.025 μg (2-fold dilution of the original 0.05 μg of dmLT); #10–0.005 μg (2-fold dilution of the original 0.01 μg of dmLT); #11–10 μg CfaEB alone (2-fold dilution); and #12- Saline (2-fold dilution). (B) SDS page gel analysis of CfaEB standards (0.1, 0.5, 1.0, and 1.5 μg) and dose formulations for Study Groups 1–12. (TIF) [file pone.0224073.s002.tif]

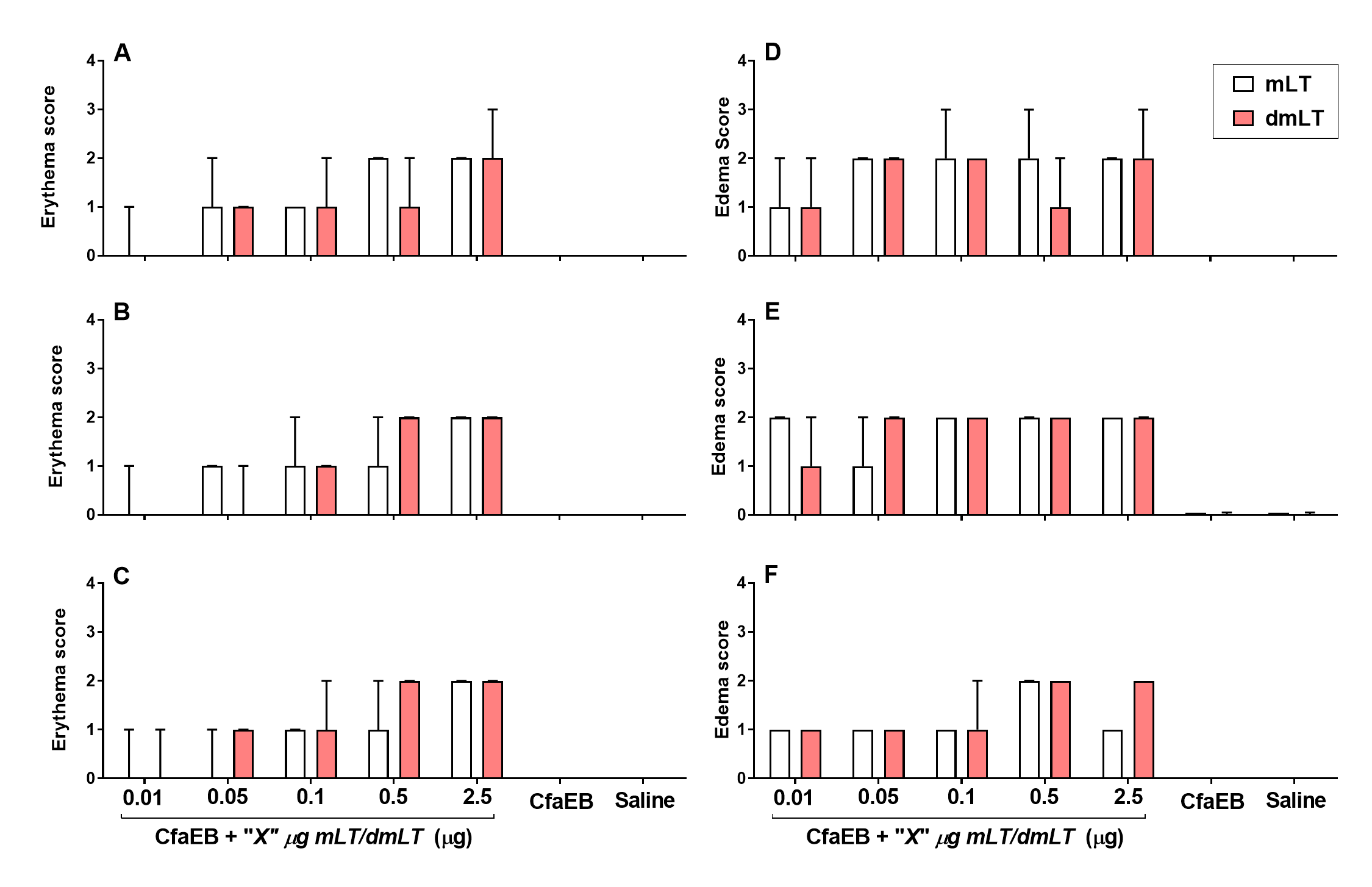

Supplement: S2 Fig — Mice were immunized with dscCfaEB and varing doses of mLT or dmLT by the ID route on days 0, 14, and 28 at sites 1, 2 and 3, respectively. Based on Adapted Draize scores (Table 2), erythema and edema at the injection sites were observed and recorded 24, 48 and 72 hours after each immunization as well as every 7 days until resolution or end of the study. Data is presented as median peak of erythema or edema ± range. (A-B-C) Erythema; (D-E-F) Edema. (TIF) [file pone.0224073.s003.tif]

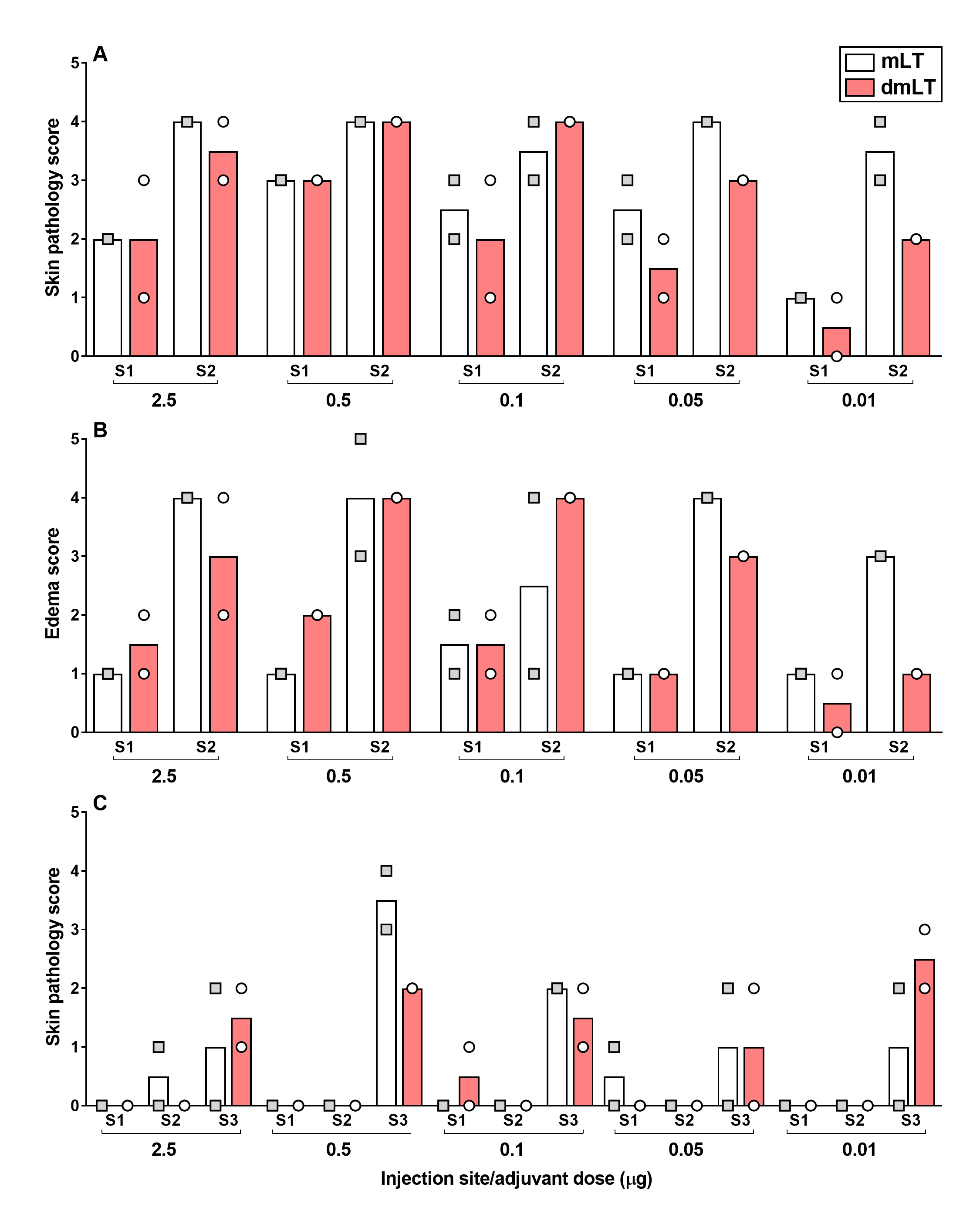

Supplement: S3 Fig — Mice were immunized with dscCfaEB and varying doses of mLT or dmLT by the ID route on days 0, 14, and 28. On day 16 of the immunization protocol, two animals from each group were euthanized and skin samples from the first (S1) and second (S2) immunizations were collected, corresponding to 16 and 2 days after each site immunization, respectively. On day 42, skin samples were collected from sites 1, 2, and 3 (S3) from two more animals, which corresponded to 42, 28, and 14 days after each site immunization, respectively. Samples were preserved and stain by hematoxylin and eosin for histopathology evaluation. The presence of edema and pathology were scored as described in the material and methods section. (A) Skin pathology scores for S1 and S2 collected on day 16. (B) Edema scores for S1 and S2 collected on day 16. (C) Skin pathology scores for S1, S2 and S3 collected on day 42. Bars represent the average score while individual values are shown as squares for mice immunized with mLT or circles for mice immunized with dmLT. (TIF) [file pone.0224073.s004.tif]
