# Supplementary material for: Evaluation of the reactogenicity, adjuvanticity and antigenicity of LT(R192G) and LT(R192G/L211A) by intradermal immunization in mice
Source: PLoS One. 2019 Nov 4;14(11):e0224073. doi: 10.1371/journal.pone.0224073 (PMC6827915; doi:10.1371/journal.pone.0224073)
Supplement: S4 Fig — (PDF) [file pone.0224073.s005.pdf]

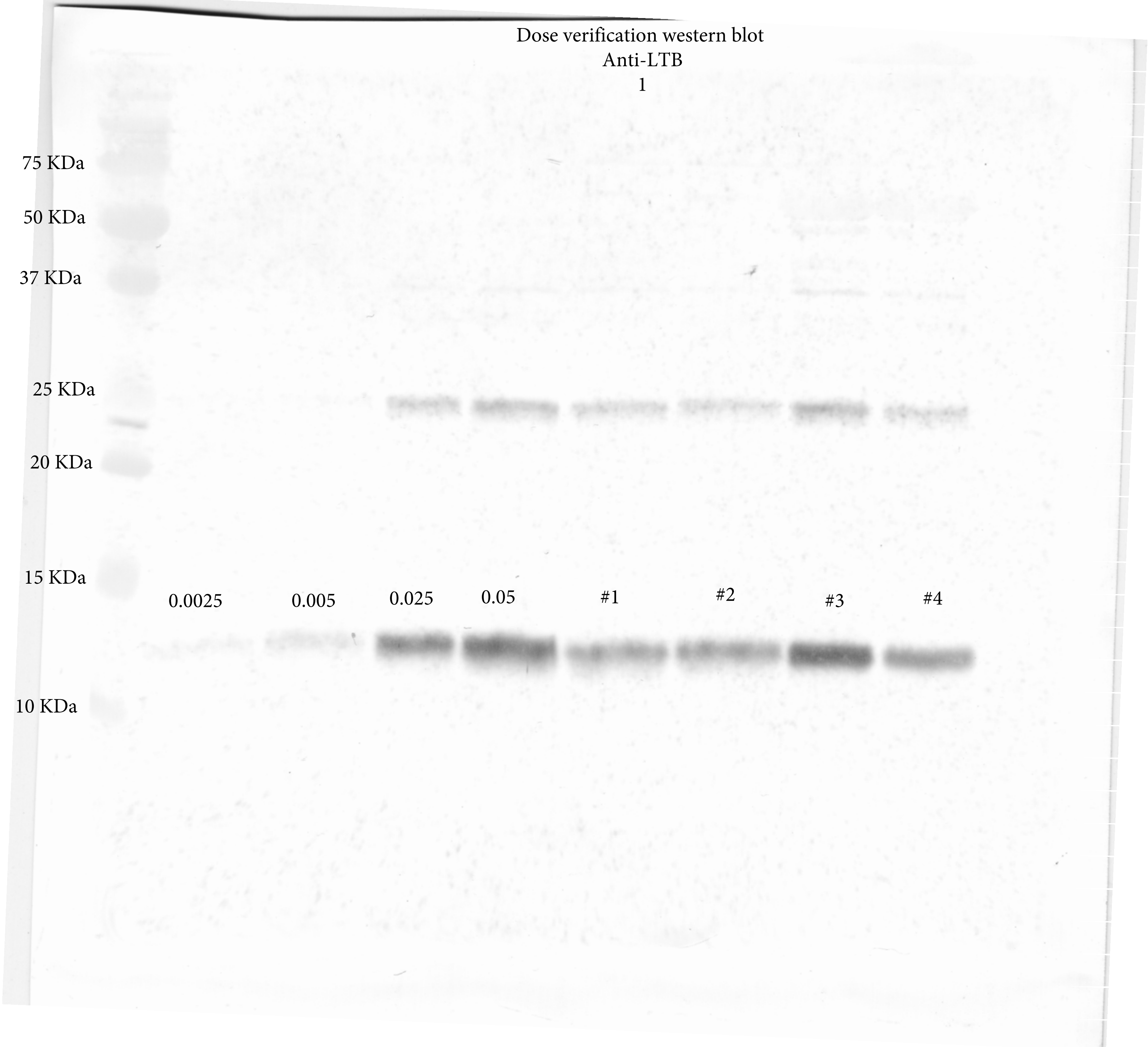
Dose verification western blot
Anti-LTB
1
75 KDa
50 KDa
37 KDa
25 KDa
20 KDa
15 KDa
10 KDa
0.0025
0.005
0.025
0.05
#1
#2
#3
#4

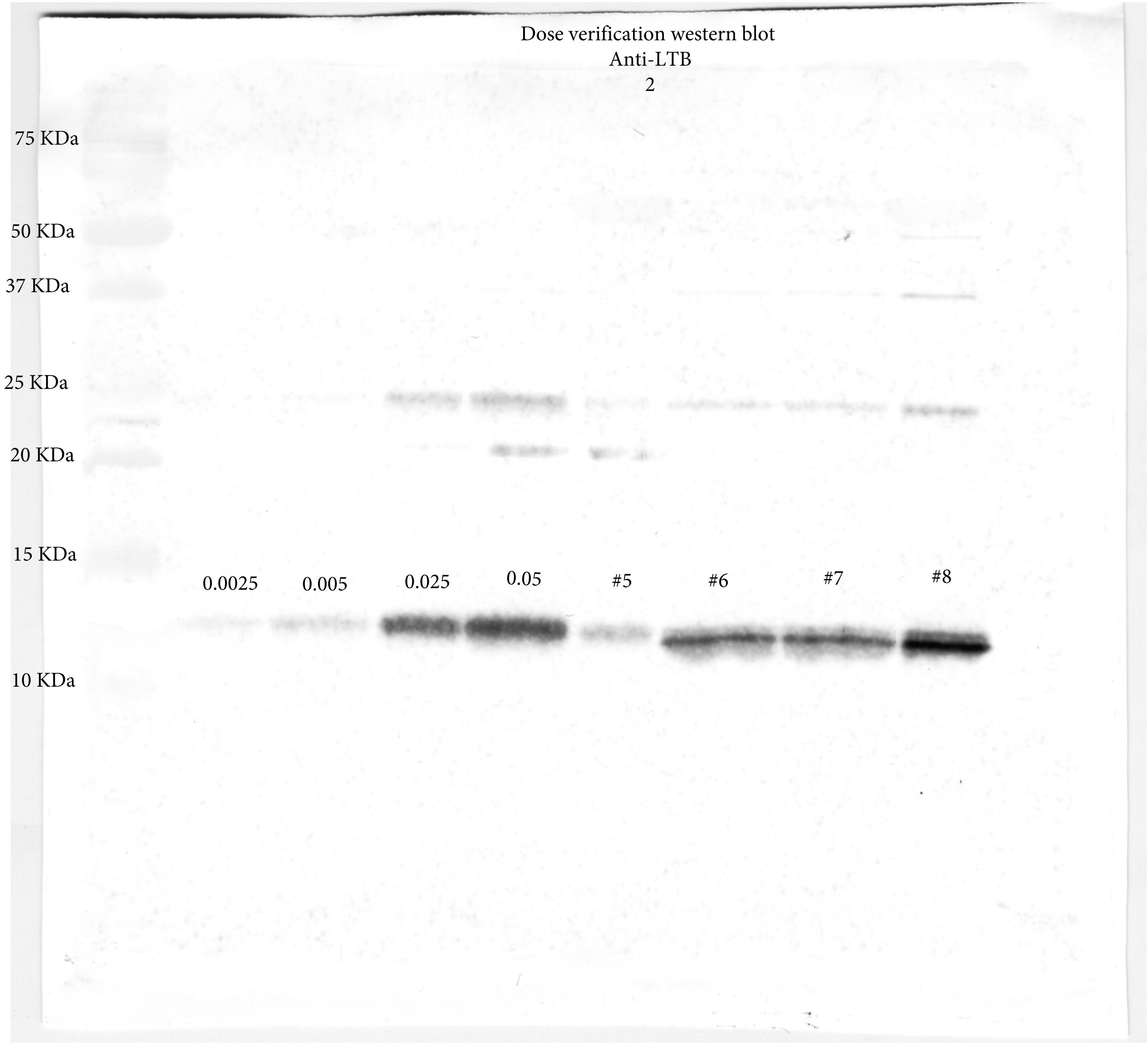
Dose verification western blot
Anti-LTB
2
75 KDa
50 KDa
37 KDa
25 KDa
20 KDa
15 KDa
10 KDa
0.0025
0.005
0.025
0.05
#5
#6
#7
#8

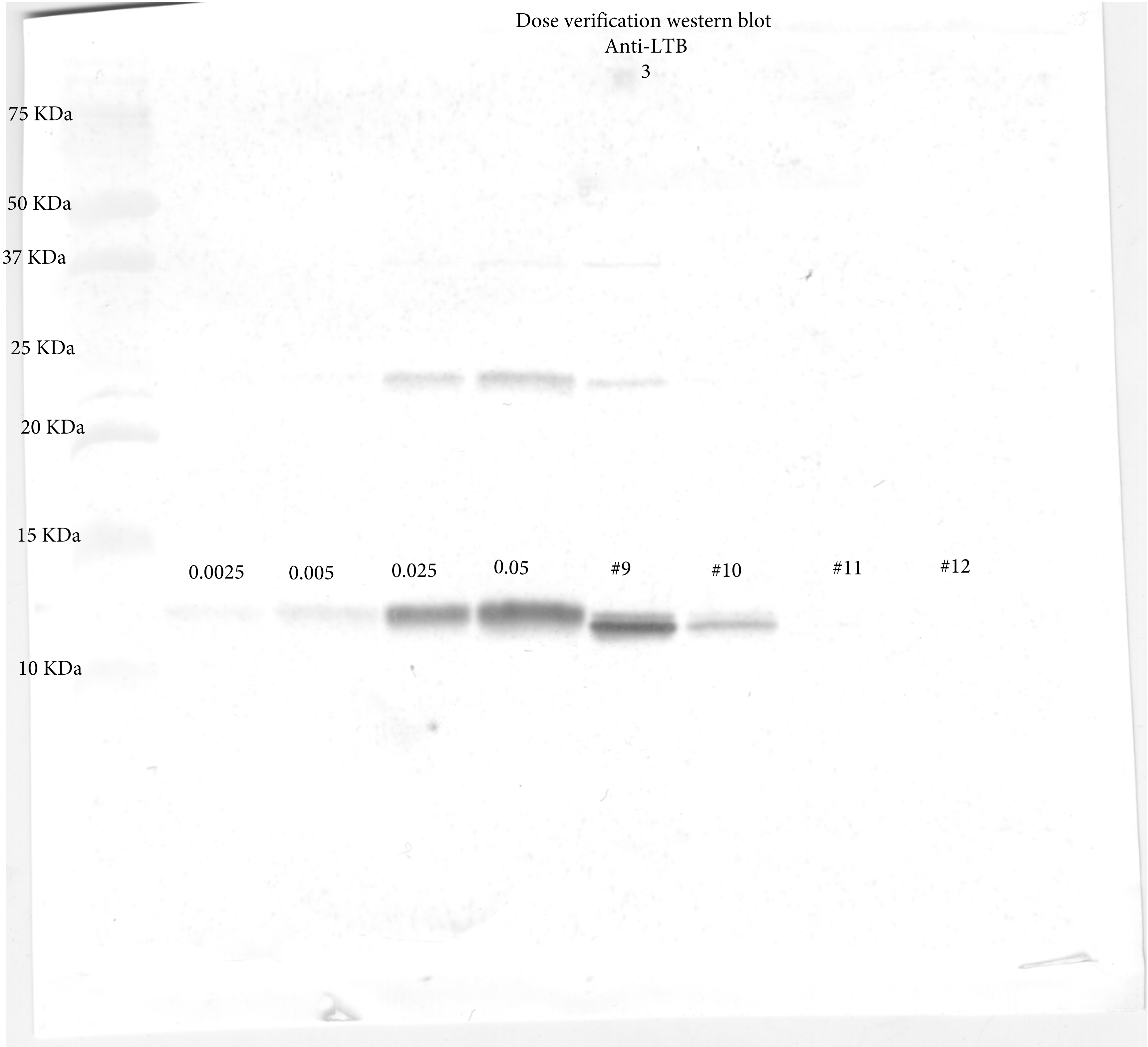
Dose verification western blot
Anti-LTB
3
75 KDa
50 KDa
37 KDa
25 KDa
20 KDa
15 KDa
10 KDa
0.0025
0.005
0.025
0.05
#9
#10
#11
#12

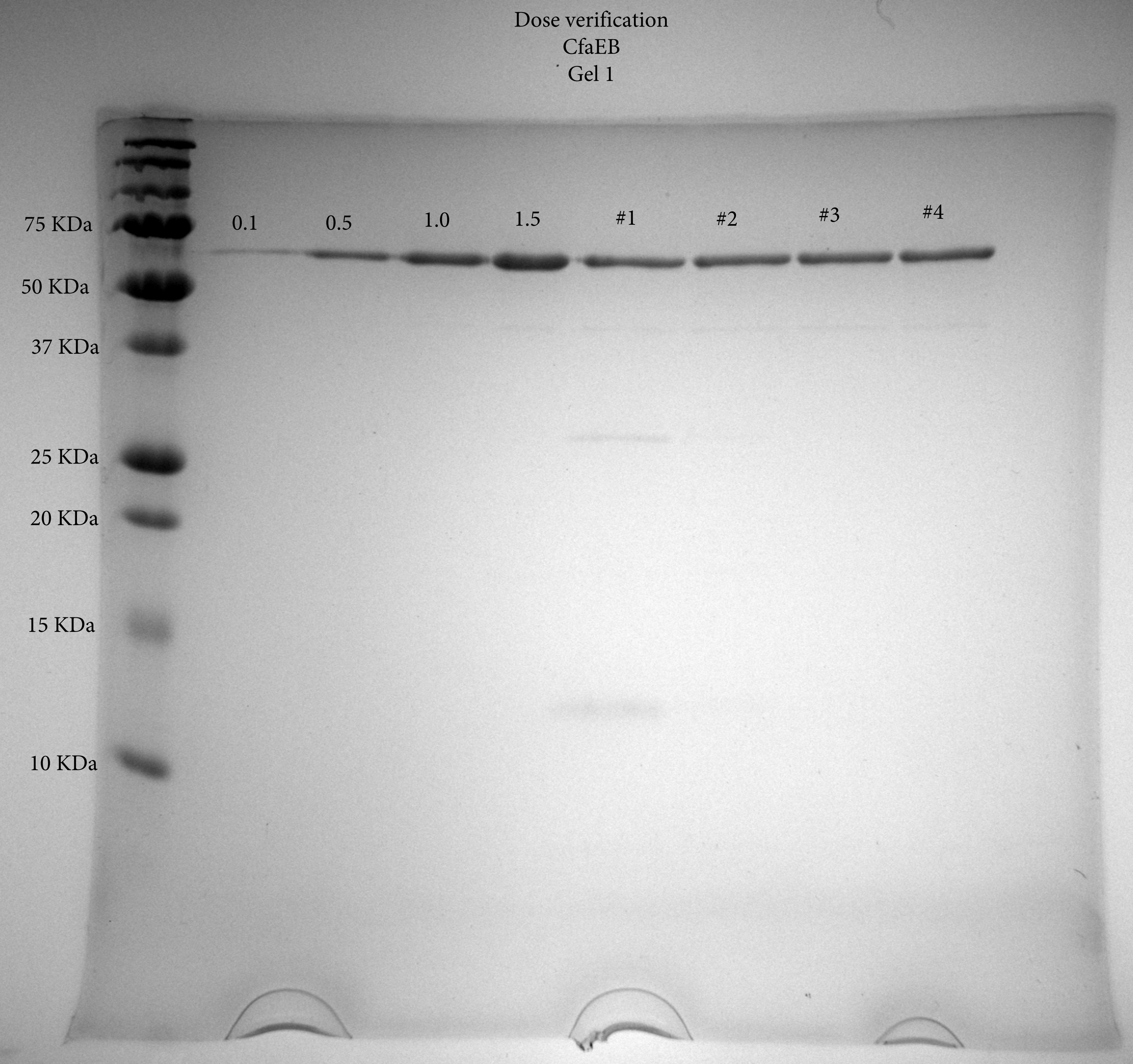
Dose verification
CfaEB
Gel 1
75 KDa
50 KDa
37 KDa
25 KDa
20 KDa
15 KDa
10 KDa
0.1
0.5
1.0
1.5
#1
#2
#3
#4

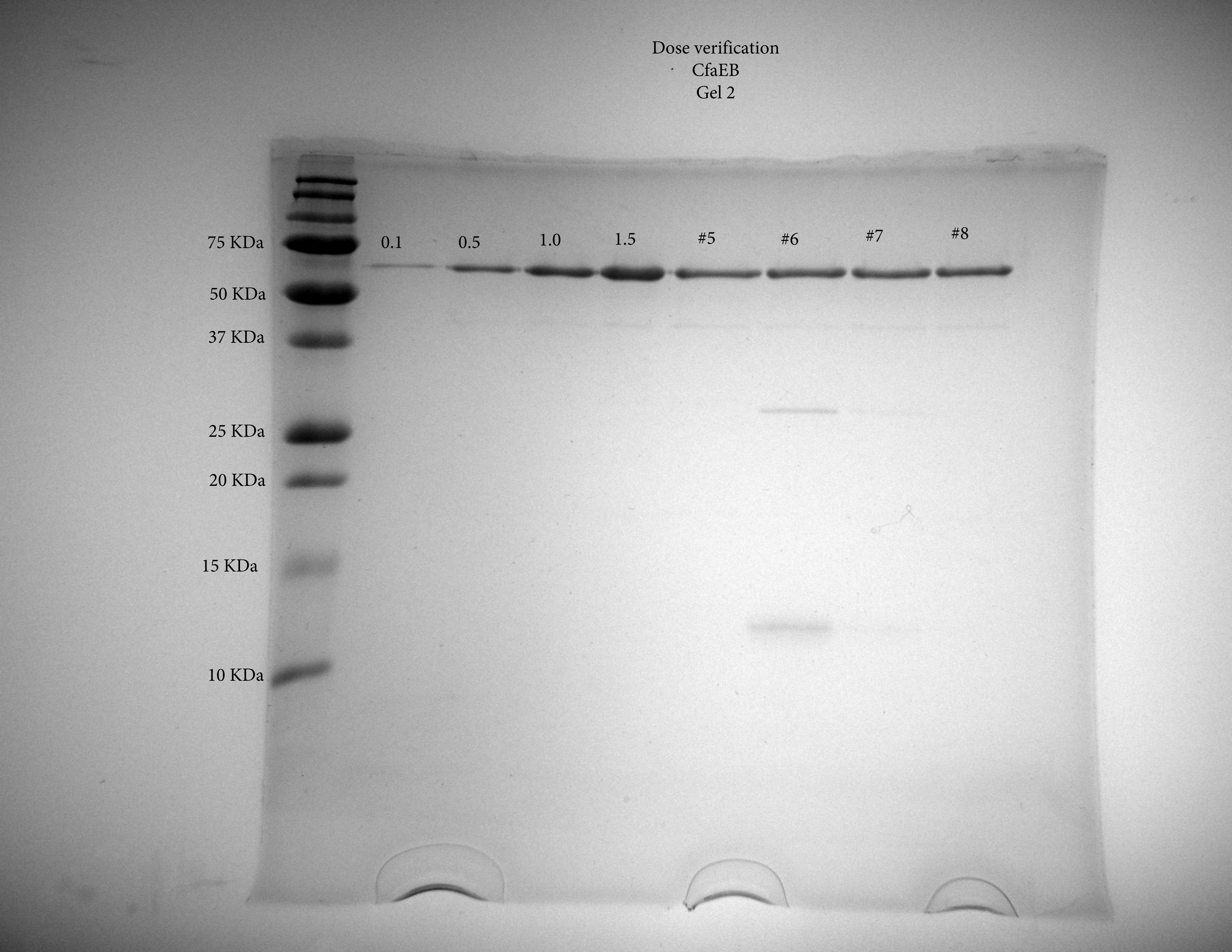
Dose verification
CfaEB
Gel 2
75 KDa
50 KDa
37 KDa
25 KDa
20 KDa
15 KDa
10 KDa
0.1
0.5
1.0
1.5
#5
#6
#7
#8

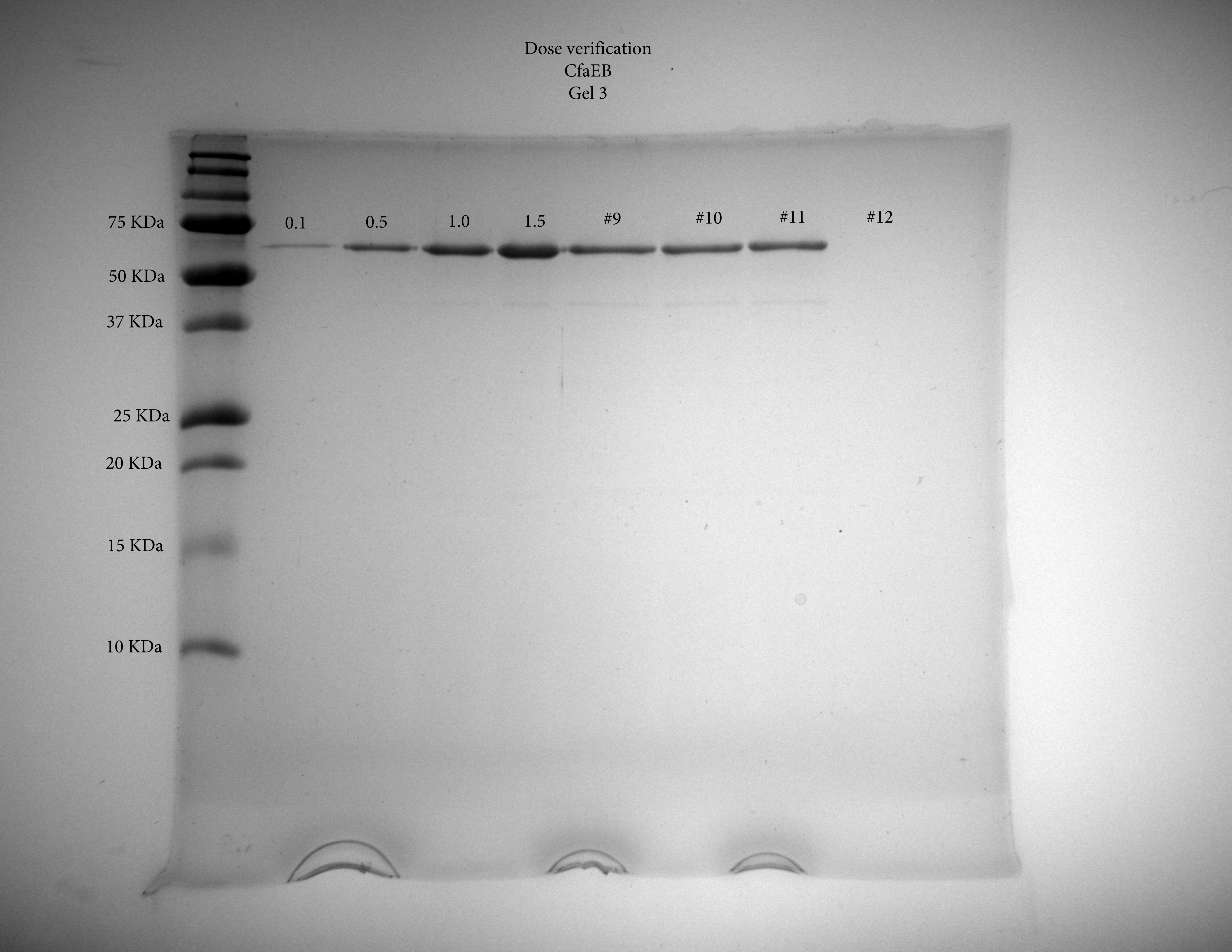
Dose verification
CfaEB
Gel 3
75 KDa
50 KDa
37 KDa
25 KDa
20 KDa
15 KDa
10 KDa
0.1
0.5
1.0
1.5
#9
#10
#11
#12
